# XIAP as a Target of New Small Organic Natural Molecules Inducing Human Cancer Cell Death

**DOI:** 10.3390/cancers11091336

**Published:** 2019-09-09

**Authors:** Diego Muñoz, Martina Brucoli, Silvia Zecchini, Adrian Sandoval-Hernandez, Gonzalo Arboleda, Fabian Lopez-Vallejo, Wilman Delgado, Matteo Giovarelli, Marco Coazzoli, Elisabetta Catalani, Clara De Palma, Cristiana Perrotta, Luis Cuca, Emilio Clementi, Davide Cervia

**Affiliations:** 1 Departamento de Química, Facultad de Ciencias, Universidad Nacional de Colombia, Bogotá 111321, Colombia; drmunozc@unal.edu.co (D.M.); agsandovalh@unal.edu.co (A.S.-H.); fhlopezv@unal.edu.co (F.L.-V.); wadelgadoa@unal.edu.co (W.D.); lecucas@unal.edu.co (L.C.); 2 Facultad de Ciencias, Universidad de Ciencias Aplicadas y Ambientales, Bogotá 111166, Colombia; 3 Tumour Cell Death Laboratory, Cancer Research UK Beatson Institute, Glasgow G61 1BD, UK; 2310786B@student.gla.ac.uk; 4 Department of Biomedical and Clinical Sciences “Luigi Sacco” (DIBIC), Università degli Studi di Milano, 20157 Milano, Italy; silvia.zecchini@unimi.it (S.Z.); matteo.giovarelli@unimi.it (M.G.); marco.coazzoli@unimi.it (M.C.); cristiana.perrotta@unimi.it (C.P.); emilio.clementi@unimi.it (E.C.); 5 Grupo de Muerte Celular, Instituto de Genética, Universidad Nacional de Colombia, Bogotá 111321, Colombia; gharboledab@unal.edu.co; 6 Department for Innovation in Biological, Agro-food and Forest systems (DIBAF), Università degli Studi della Tuscia, 01100 Viterbo, Italy; ecatalani@unitus.it; 7 Unit of Clinical Pharmacology, University Hospital “Luigi Sacco”-ASST Fatebenefratelli Sacco, 20157 Milano, Italy; clara.depalma@asst-fbf-sacco.it; 8 Scientific Institute IRCCS “Eugenio Medea”, 23842 Bosisio Parini, Italy

**Keywords:** phytochemicals, small organic agents, *Piper eriopodon*, alkenylphenols, human cancer cells, cell death, apoptosis, caspase-independent cell death, XIAP antagonists, XIAP-BIR3 domain

## Abstract

X-linked inhibitor of apoptosis protein (XIAP) is an emerging crucial therapeutic target in cancer. We report on the discovery and characterisation of small organic molecules from *Piper* genus plants exhibiting XIAP antagonism, namely erioquinol, a quinol substituted in the 4-position with an alkenyl group and the alkenylphenols eriopodols A–C. Another isolated compound was originally identified as gibbilimbol B. Erioquinol was the most potent inhibitor of human cancer cell viability when compared with gibbilimbol B and eriopodol A was listed as intermediate. Gibbilimbol B and eriopodol A induced apoptosis through mitochondrial permeabilisation and caspase activation while erioquinol acted on cell fate via caspase-independent/non-apoptotic mechanisms, likely involving mitochondrial dysfunctions and aberrant generation of reactive oxygen species. In silico modelling and molecular approaches suggested that all molecules inhibit XIAP by binding to XIAP-baculoviral IAP repeat domain. This demonstrates a novel aspect of XIAP as a key determinant of tumour control, at the molecular crossroad of caspase-dependent/independent cell death pathway and indicates molecular aspects to develop tumour-effective XIAP antagonists.

## 1. Introduction

The characterisation of small molecules (whose molecular weight does not exceed 900 Daltons) with well-defined chemical structures is a good approach to develop new therapeutic agents in proliferative, infectious, or neurodegenerative disorders [1,2,3,4,5]. Natural products possess enormous structural and chemical diversity that cannot be matched by any synthetic libraries of small molecules and continue to show a great translational potential [6,7,8,9,10]. In some cases, the complex chemical composition of some natural products has made difficult their isolation, structure elucidation and characterisation, thus prompting the search of new efficient synthetic pathways. In recent years the interest in the fundamental understanding of natural products and their engineered variants has been strongly renewed [6].

The simple active chemical structures of phenolic compounds from plants make them optimal lead candidates because of their broad biological activity, especially the protective, anti-oxidant and anti-tumour effects [11,12,13,14]. Plants of the genus *Piper* (Piperaceae family), are a very common food resource in neotropical forests and are widely used to obtain culinary spices. *Piper* genus constitutes one major class of medicinal plants and contains a valuable resource of phenolic bioactive compounds [15,16,17,18,19,20,21]. Among them, piplartine, hydroxychavicol, 4-nerodlidylcatechol and gibbilimbols A–D displayed potent cytotoxic/anti-tumoural effects in a variety of human cancer cells in vitro and in vivo [19,22,23,24,25,26,27,28,29].

Apoptosis, a closely regulated programmed cell death mechanism, is an essential process to maintain tissue homeostasis and its escape it is one of the hallmarks of cancer [30]. Substantial advances have been made on apoptosis-based anti-cancer therapeutics [31]. The most potent human IAP currently identified is the X-linked inhibitor of apoptosis protein (XIAP), a 57 kDa protein with three zinc-binding baculovirus IAP repeat (BIR) domains (BIR 1–3) which may also have actions additional to regulation of apoptosis [32]. The anti-apoptotic function of XIAP is antagonised by the second mitochondria-derived activator of caspases or direct IAP binding protein with low pI (Smac/DIABLO), a mitochondria protein released during apoptosis. The key role of XIAP and its potential clinical relevance is well established in tumours and several XIAP inhibitors have been developed or discovered as cytotoxic agents [32,33,34,35,36,37,38,39,40,41,42,43]. Despite different small molecules that inhibit XIAP have been identified and are moving through the pipeline of clinical development, the need of new ones to refine further therapeutic approaches based on XIAP antagonism is undeniable in translational research [41].

Herein we wish to report the discovery and chemical/biological characterisation of novel natural small compounds from *Piper* genus. Furthermore, a deeper insight into their cell death mechanism in human cells provides a proof-of-concept study of their pharmaceutical potential as antagonists of XIAP that may open important insights on XIAP as a suitable turning point for multiple cellular pathways.

## 2. Results and Discussion

### 2.1. Structural Identification of New Piper Genus-Derived Compounds

**Figure 1:**
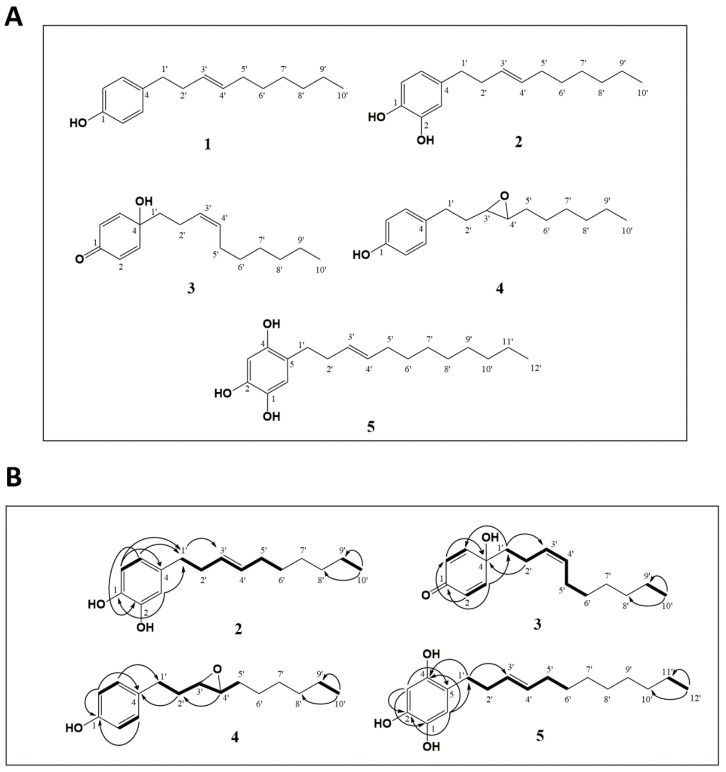
Identification of new *Piper* genus-derived compounds. (**A**) Structures of compounds 1–5. (**B**) Key correlated spectroscopy (COSY) (bold) and heteronuclear multiple bond correlation (HMBC) (H→C) for compounds **2–5**.

The chemical structures of compounds isolated from leaves of *P. eriopodon* (Figure 1A) were identified by interpretation of their corresponding high resolution electrospray ionisation mass spectrometry (HRESIMS), ^1^H- and ^13^C-NMR (nuclear magnetic resonance) spectral data, including attached proton test (APT), correlated spectroscopy (COSY), heteronuclear multiple quantum coherence (HMQC) and heteronuclear multiple bond correlation (HMBC) experiments, as well as by comparison of the spectral data with those reported in the literature. 

Compound 1 (Figure S1, Tables S1 and S2) was obtained as colorless oil and identified unequivocally as gibbilimbol B ((*E*)-4-(dec-3′-enyl)phenol) [19]. 

Compound 2 (Figure S2, Tables S1 and S2) was obtained as pale yellow oil. The molecular formula for compound **2** was established as C_16_H_24_O_2_ based on the HRESIMS peak at *m*/*z* 247.1706 [M-H]^−^ (calcd. 247.1703). The ^1^H- NMR spectrum showed clear signals for a 1,2,4-trisubstituted aromatic ring δH 6.77 (1H, d, *J* = 7.6 Hz, H-6), 6.71 (1H, s, H-3), 6.60 (1H, d, *J* = 7.5 Hz, H-5) and an alkenyl fragment. The ^13^C-NMR spectrum showed ten signals, practically the same as the alkenyl chain of gibbilimbol B, including the double bond position in C-3′, which was confirmed by correlations observed in both COSY and HMBC experiments (Figure 1B). Based on the ^13^C-NMR chemical shifts of the allylic carbons δ_C_ 34.6 (C-2′) and δ_C_ 32.6 (C-5′), the configuration of the double bond for compound 2 was assigned as *E* [18], by comparison with the ^13^C-NMR chemical shift of the allylic carbons in the *E* analogue gibbilimbol B (δ_C_ 34.6 (C-2′) and δ_C_ 32.6 (C-5′)), which differed significantly from the chemical shift values reported for the *Z* analogue climacostol [δ_C_ 33.2 (C-1′) and δ_C_ 27.3 (C-4′)] [44]. Thus, the chemical structure of compound **2** was elucidated as (*E*)-4-(dec-3′-enyl)benzene-1,2-diol and it was given the common name of eriopodol A.

Compound **3** (Figure S3, Tables S1 and S2) was obtained as clear oil and its molecular formula was deduced as C_16_H_24_O_2_ from the HRESIMS spectrum, which exhibited a molecular ion peak at *m*/*z* 247.1706 [M-H]^−^ (calcd. 247.1703). The ^1^H-NMR spectrum for compound 3 showed signals for an alkenyl chain and two signals in δH 6.11 (2H, d, *J* = 9.94 Hz) and 6.81(2H, d, *J* = 9.96 Hz). The ^13^C-NMR spectrum for compound **3** showed signals for an α-β unsaturated carbonyl in δ_C_ 185.9, an oxygenated quaternary carbon in δ_C_ 69.6 and ten signals for the typical side chain of the alkenyl fragment. Based on the correlations observed in COSY and HMBC experiments (Figure 1B), the structure of 3 was determined as a quinol derivative, substituted in the 4-position with an alkenyl group. The position and geometry of the double bond of compound 3 was assigned by comparing the chemical shift values of the allylic carbons δ_C_ 32.4 (C-2′) and δ_C_ 26.6 (C-5′) as explained above for eriopodol A. The geometry of compound *3* was determined as *Z* and its chemical structure was elucidated as (*Z*)-4-(dec-3′-enyl)-4-hydroxycyclohexa-2,5-dien-1-one. The common name of erioquinol was then assigned.

Compound 4 (Figure S4, Tables S1 and S2) was obtained as pale yellow oil. The molecular formula for compound 4 was confirmed to be C_16_H_24_O_2_ based on the HRESIMS peak at *m*/*z* 247.1715 [M-H]^−^ (calcd. 247.1703). The ^1^H-NMR and ^13^C-NMR spectra of compound 4 showed almost the same chemical shifts as the alkenylphenol gibbilimbol B, but without the unsaturated signal in the ^1^H-NMR spectrum. Therefore, the carbons C-3′ and C-4′ showed chemical shifts in δ_C_ 59.6 (C-3′) and δ_C_ 58.6 (C-4′), corresponding two oxygenated methines from an epoxide group, which was confirmed with COSY and HMBC experiments (Figure 1B). The structure of compound **4** was elucidated as 4-(3′,4′-epoxydecenyl)phenol and the common name of eriopodol B was assigned.

Compound 5 (Figure S5, Tables S1 and S2) was obtained as pale yellow amorphous solid (m.p. 138.5 °C). The molecular formula for compound **5** was established as C_18_H_28_O_3_ based on the HRESIMS peak at *m*/*z* 291.1973 [M-H]^−^ (calcd. 291.1966). The NMR data for compound **5** were very close to those of eriopodol A, although it contains one additional hydroxyl group in the benzene ring and two additional carbons at the end of the alkenyl chain (Figure 1B). The position and geometry of the double bound for compound **5** was assigned as explained above. The structure of compound **5** was elucidated as (*E*)-5-(dodec-3′-enyl)benzene-1,2,4-triol and the common name of eriopodol C was assigned.

Taken together, phytochemical investigation of leaves from P. eriopodon yielded four new alkenyl derivatives and one known compound. In particular, erioquinol is a new quinol substituted in the 4-position with an alkenyl group and eriopodols A-C correspond to new alkenylphenols. The known isolated compound was originally identified as gibbilimbol B, from the medicinal plant P. gibbilimbum [19] and, more recently, from P. malacophyllum [21] and P. eriopodon [29]. The simple chemical structure of alkenylphenols are characterised by hydroxylated benzenes, substituted by side alkyl chains of different lengths with at least one double bond, generally with E geometry. Alkenylphenols with different reported biological properties, such as antibacterial, anti-parasitic, anti-inflammatory and cytotoxic activities, are widely found in the Piper genus [17,19,20,21,45]. Quinols are 4-hydroxycyclohexa-2,5-dien-1-ones which rarely occur as derivatives of some natural products [46,47,48]. An important feature of quinols substituted in the 4-position with aryl groups, is that they represent a class of potent anti-tumour molecules with activities against colon, renal, and breast cancer cells [49,50,51].

### 2.2. Piper Genus-Derived Compounds Exhibit Cytotoxic Effects 

**Figure 2:**
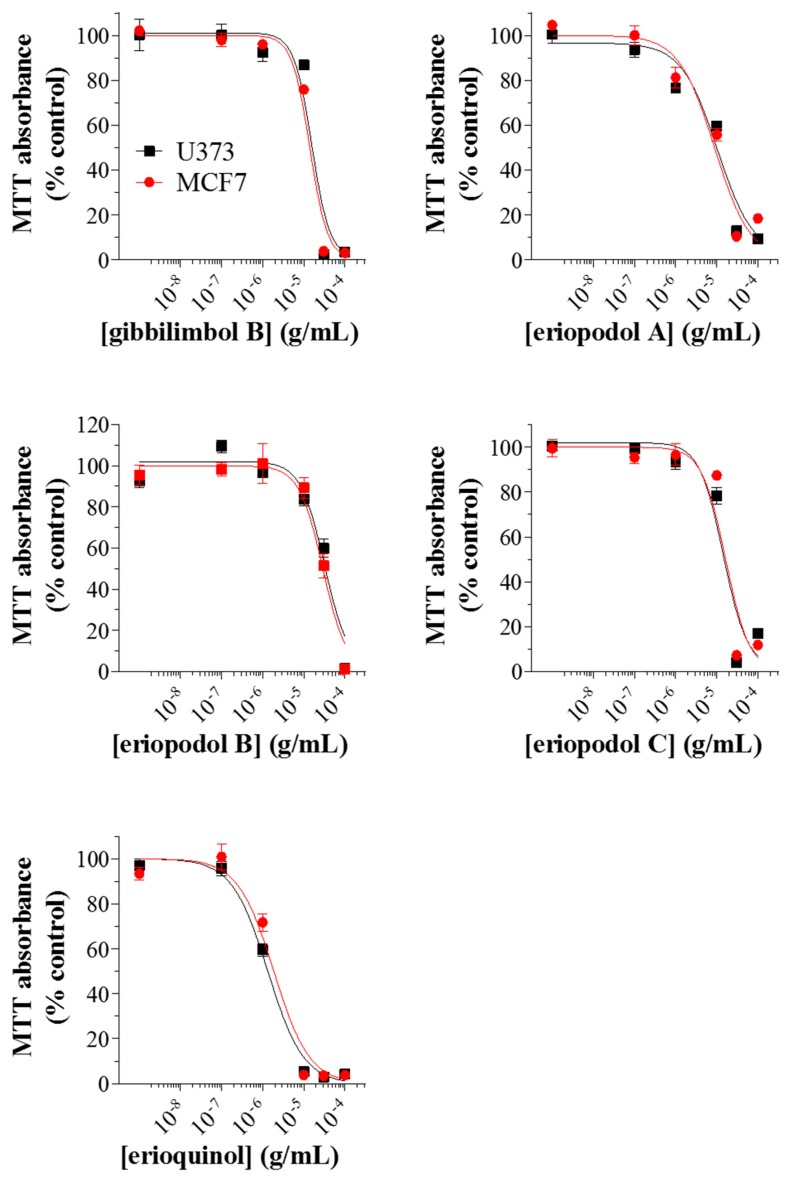
*Piper* genus-derived compounds exhibit cytotoxic effects in human cancer cells. U373 and MCF7 cells were treated with increasing concentrations of gibbilimbol B, eriopodol A, eriopodol B, eriopodol C, and erioquinol for 24 h before 3-(4,5-dimethylthiazol-2-yl)-2,5-diphenyltetrazolium bromide (MTT) assay. Results are expressed by setting the absorbance of the reduced MTT in the respective control (vehicle-treated) samples, i.e., absence of compounds, as 100%. The data points are representative of four independent experiments.

Several recent studies in glioblastoma and breast cancer cells have reported that extracts or active compounds isolated from *Piper* genus possess anti-tumoural/pro-apoptotic properties [52,53,54,55,56,57,58,59,60,61]. In order to assess whether the compounds we isolated could be developed further for therapeutic applications, we tested their cytotoxic action in the human cancer cells, U373 (glioblastoma astrocytoma) and MCF7 (breast adenocarcinoma) cell lines, since they are widely used as suitable in vitro models of cancer research. We first examined the effects of gibbilimbol B, eriopodols A–C, and erioquinol on cell viability. Gibbilimbol B was used as a reference compound of *Piper* genus derivatives, since its cytotoxic action has been previously tested in various tumour cells, including MCF7 [19,29]. In our experiments, cell viability was analysed by 3-(4,5-dimethylthiazol-2-yl)-2,5-diphenyltetrazolium bromide (MTT) assay after treatment with previously mentioned compounds at increasing concentrations for 24 h. As shown in Figure 2, a concentration-dependent inhibition of MTT absorbance was observed for all compounds with an IC_50_ (the concentration producing half the maximum inhibition) ranging from 1.78 to 31.91 μg/mL; the rank order of potencies was: erioquinol > eriopodol A > eriopodol C > gibbilimbol B > eriopodol B and erioquinol > eriopodol A > eriopodol C/gibbilimbol B > eriopodol B for U373 and MCF7 cells, respectively (Table 1). Their effects were maximal (E_max_—concentration producing the maximum effect—nearly 100% inhibition) between 10–100 µg/mL. 

**Figure 3:**
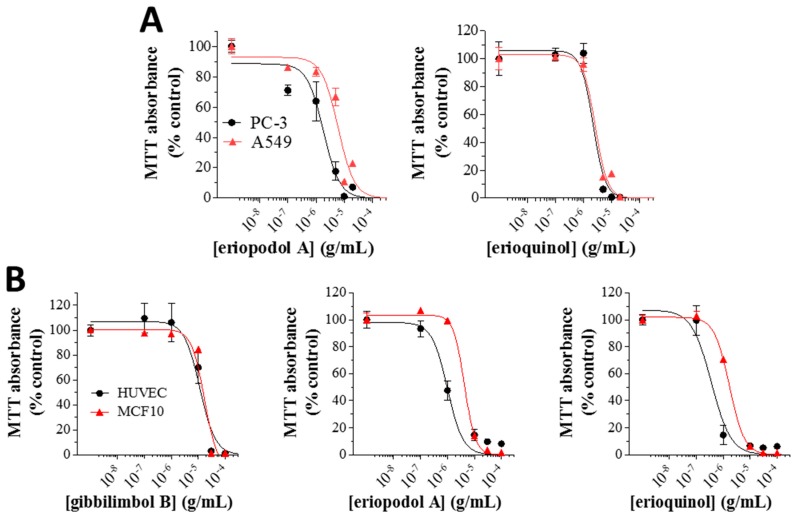
*Piper* genus-derived compounds exhibit cytotoxic effects in cancer and non-transformed human cells. (**A**) PC-3/A549 cells were treated with increasing concentrations of eriopodol A and erioquinol while (**B**) human umbilical vein endothelial cells (HUVEC)/MCF10 cells were treated with increasing concentrations of gibbilimbol B, eriopodol A, and erioquinol, for 24 h before 3-(4,5-dimethylthiazol-2-yl)-2,5-diphenyltetrazolium bromide (MTT) assay. Results are expressed by setting the absorbance of the reduced MTT in the respective control (vehicle-treated) samples, i.e., absence of compounds, as 100%. The data points are representative of four independent experiments.

Eriopodol A and erioquinol were selected for further investigation, as they displayed the most potent inhibitory effects on cell viability. Gibbilimbol B (available in high quantity) was also included. When compared with gibbilimbol B [29], the higher cytotoxic effect of eriopodol A and erioquinol (24 h), was also shown by MTT assays using additional cell lines, like human A549 lung (IC_50_ of eriopodol A and erioquinol: 6.12 and 2.65 μg/mL, respectively) and PC-3 prostate (IC_50_ of eriopodol A and erioquinol: 1.84 and 2.21 μg/mL, respectively) cancer cells, further confirming enhanced pharmacological activity of these new *Piper* genus derivatives (Figure 3A). 

Similar results were obtained in human umbilical vein endothelial cells (HUVEC) (IC_50_ of 24 h gibbilimbol B, eriopodol A, and erioquinol: 11.49, 0.99, and 0.36 μg/mL, respectively) and the non-tumourigenic human breast MCF10 cells (IC_50_ of 24 h gibbilimbol B, eriopodol A, and erioquinol: 17.11, 4.27, and 1.70 μg/mL, respectively) (Figure 3B). The fact that the potency of the compounds was even slightly higher in these non-transformed/high proliferating cells suggests that their effects are not necessarily correlated to the cancerous origin of cells, in agreement with other small molecules we have recently characterised [62]. On the other hand, many cytotoxic compounds, including chemotherapy agents, are specifically designed to primarily affect rapidly proliferating cells, and many “normal” cells are also highly proliferative, such as cells in the bone marrow. The possibility that *Piper* genus-derived compounds preferentially affect high proliferating vs. low proliferating cells remains to be elucidated.

**Figure 4:**
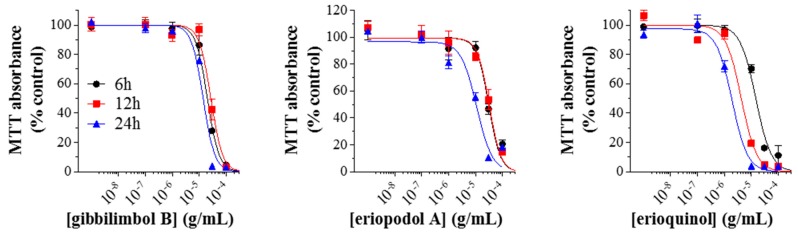
Time-response of *Piper* genus-derived compounds on cell viability. MCF7 cells were treated with increasing concentrations of gibbilimbol B, eriopodol A, and erioquinol for 6, 12, and 24 h before 3-(4,5-dimethylthiazol-2-yl)-2,5-diphenyltetrazolium bromide (MTT) assay. Results are expressed by setting the absorbance of the reduced MTT in the respective control (vehicle-treated) samples, i.e., absence of compounds, as 100%. The data points are representative of four independent experiments.

We then measured the concentration-dependent inhibition of MTT absorbance at increasing times of exposure in MCF7 cells, used as reference cell line. Our results indicated that the potency of gibbilimbol B did not substantially change (IC_50_-6 h: 20.31 µg/mL; 12 h: 27.36 µg/mL; 24 h: 16.44 µg/mL) while the potency of eriopodol A increased at 24 h (IC_50_ - 6 h: 31.19 µg/mL; 12 h: 32.75 µg/mL; 24 h: 11.13 µg/mL) (Figure 4). Of interest, the potency of erioquinol was greater than gibbilimbol B and eriopodol A at each time-point, even increasing over time (IC_50_-6 h: 14.72 µg/mL; 12 h: 4.25 µg/mL; 24 h: 1.93 µg/mL). These comparative data indicate that erioquinol is the most potent compound with faster kinetics when compared with gibbilimbol B; eriopodol A has a somewhat intermediate behavior.

### 2.3. Piper Genus-Derived Compounds Induce Cell Death

**Figure 5:**
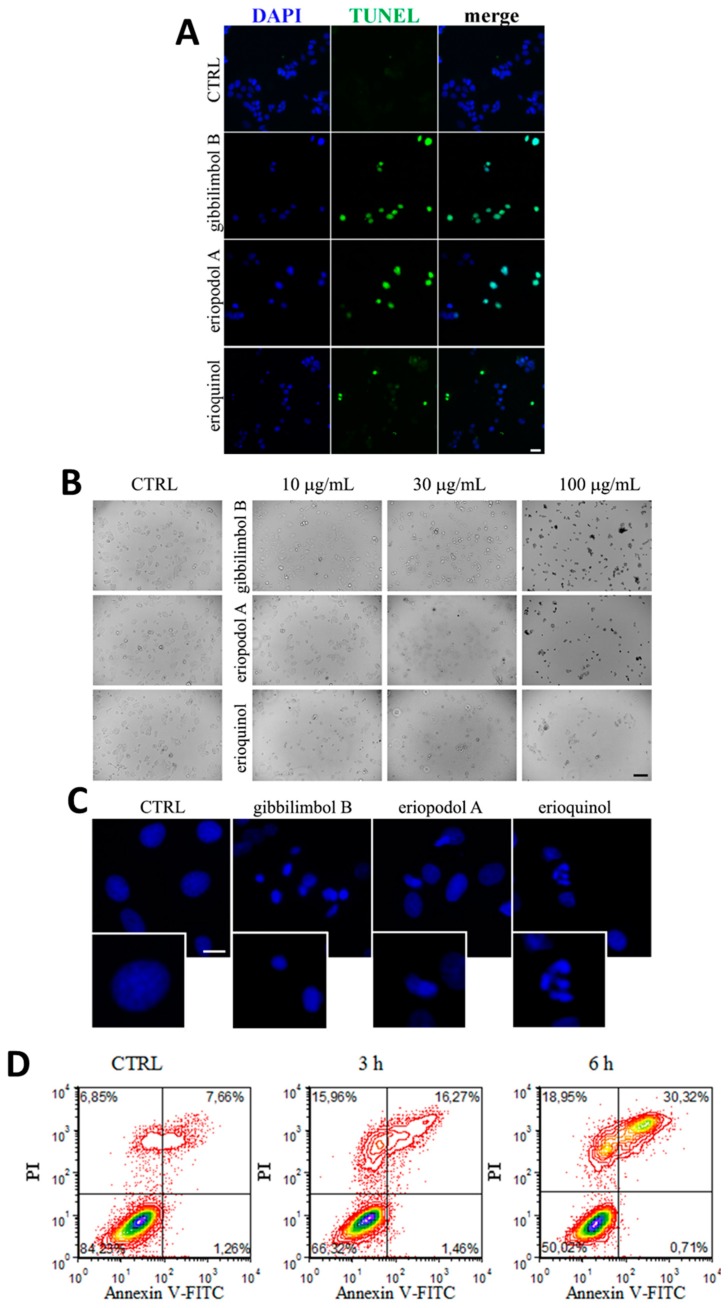
*Piper* genus-derived compounds induce cell death. (**A**) terminal deoxynucleotidyl transferase dUTP nick end labeling (TUNEL) staining of MCF7 cells treated for 12 h in the absence (CTRL, control) and in the presence of gibbilimbol B/eriopodol A (30 µg/mL) or erioquinol (10 µg/mL). 4’,6-diamidine-2’-phenylindole dihydrochloride (DAPI) was used for nuclei detection. Scale bar = 50 µm. (**B**) Bright field microscopy of MCF7 cells treated for 6 h in the absence (CTRL) and in the presence of gibbilimbol B, eriopodol A, or erioquinol at increasing concentrations. Scale bar = 100 µm. (**C**) 4’,6-diamidine-2’-phenylindole dihydrochloride (DAPI) staining of MCF7 cells treated for 6 h in the absence (CTRL) and in the presence of gibbilimbol B/eriopodol A (30 µg/mL) or erioquinol (10 µg/mL). Scale bar = 10 µm. Lower panels represent enlarged image details. (**D**) Evaluation by flow cytometry of Annexin V-fluorescein isothiocyanate(FITC)/propidium iodide (PI) staining in MCF7 cells treated in the absence (CTRL) and in the presence of 10 µg/mL erioquinol, for 3 and 6 h. Quadrants are drawn, and relative proportion of labelled cells is indicated. The events shown in the lower left-hand quadrant are unlabeled cells. Images and data are representative of four independent experiments.

MCF7 cells treated for 12 h with gibbilimbol B and eriopodol A (30 µg/mL) showed an inter-nucleosomal degradation of genomic DNA typical of late apoptotic cells, as determined by a terminal deoxynucleotidyl transferase dUTP nick end labeling (TUNEL) assay (Figure 5A), while DNA fragmented cells were few following erioquinol (10 µg/mL) treatment. Bright field microscopy demonstrated that cells exposed to increasing concentrations of gibbilimbol B and eriopodol A at 6 h (a temporal window sufficient to determine their cytotoxic effects) had morphological hallmarks of apoptosis, such as progressive roundness, shrunken cytoplasm and the formation of condensed nuclei (Figure 5B). In contrast, cells treated with erioquinol displayed a translucent cytoplasm and no overall nuclei condensation. Of interest, 4’,6-diamidine-2’-phenylindole dihydrochloride (DAPI) staining clearly revealed the nuclei of cells undergoing apoptosis in the presence of gibbilimbol B and eriopodol A (30 µg/mL) for 6 h, while erioquinol (10 µg/mL) treatment was associated with the appearence of multinucleated cells (Figure 5C). Accordingly, when analysed by flow cytometry using Annexin V and propidium iodide (PI) staining, MCF7 cells treated with erioquinol showed a progressive and marked increase of membrane disruption, as shown by early positivity to both Annexin V and PI staining, while the typical early apoptotic pattern, evidenced as Annexin V^+^/PI^−^ was almost undetectable over time (Figure 5D). 

**Figure 6:**
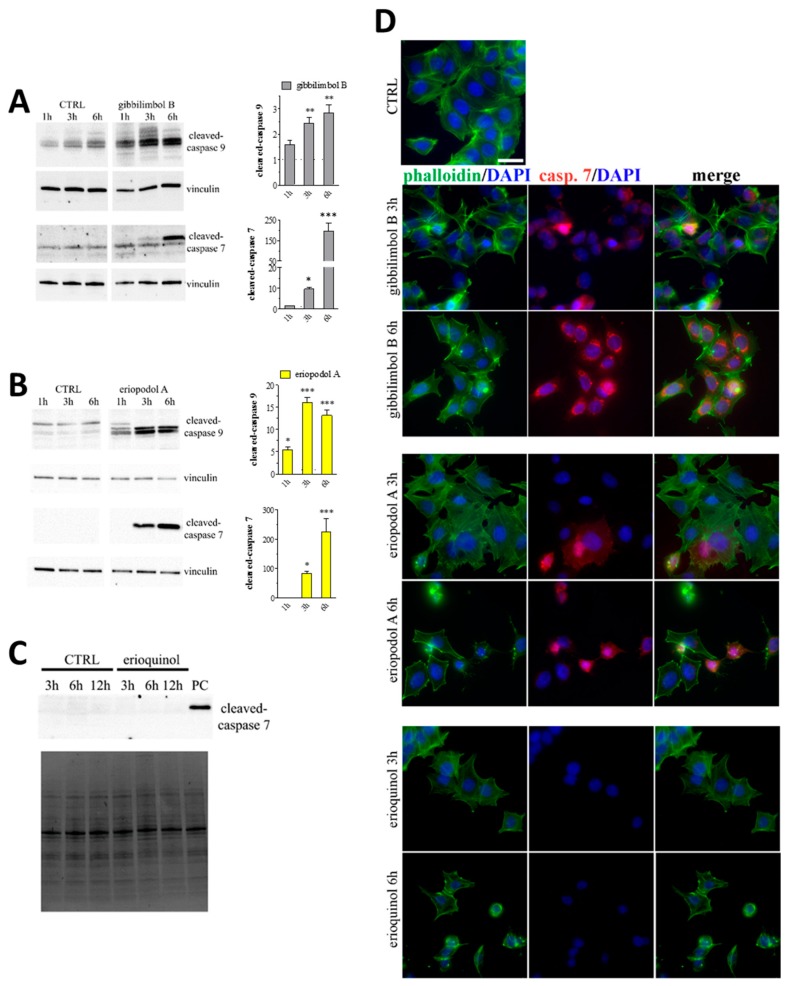
*Piper* genus-derived compounds induce cell death. Western blot analysis of cleaved-caspase 9 and 7 in MCF7 cells treated for increasing times in the absence (CTRL, control) and in the presence of (**A**) gibbilimbol B or (**B**) eriopodol A. Vinculin was used as internal standard. Right panels: densitometric analysis expressed as fold change of CTRL. Images and data are representative of three-five independent experiments. * *p* < 0.01, ** *p* < 0.001, and *** *p* < 0.0001 relative to CTRL. (**C**) Western blot analysis of cleaved-caspase 7 in MCF7 cells treated for increasing times in the absence (CTRL) and in the presence of 10 µg/mL erioquinol. The stain-free gel was used as loading control. Images are representative of three independent experiments. PC: positive control. (**D**) Immunofluorescence imaging of cleaved-caspase 7 (punctate red pattern) in MCF7 cells treated for 3 and 6 h in the absence (CTRL) and in the presence of gibbilimbol B/eriopodol A (30 µg/mL) or erioquinol (10 µg/mL). 4’,6-diamidine-2’-phenylindole dihydrochloride (DAPI) (blue) and phalloidin (green) were used for nuclei and cytoskeleton detection, respectively. Images are representative of four independent experiments. Scale bar = 25 µm.

**Figure 7:**
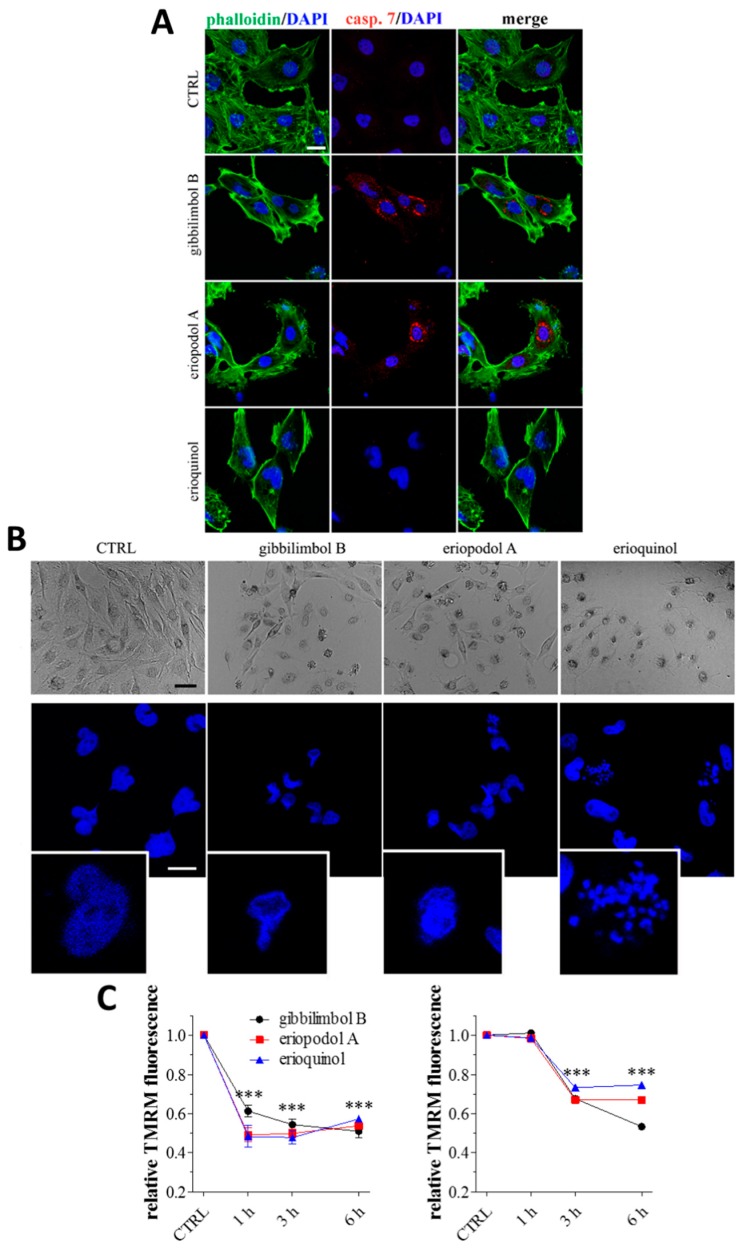
*Piper* genus-derived compounds induce cell death and mitochondrial dysfunction. (**A**) Immunofluorescence (confocal) imaging of cleaved-caspase 7 (punctate red pattern) in U373 cells treated for 6 h in the absence (CTRL, control) and in the presence of gibbilimbol B/eriopodol A (30 µg/mL) or erioquinol (10 µg/mL). 4’,6-diamidine-2’-phenylindole dihydrochloride (DAPI) (blue) and phalloidin (green) were used for nuclei and cytoskeleton detection, respectively. Scale bar = 25 µm. (**B**) Bright field microscopy (upper panels) and DAPI staining (lower panels) of U373 cells treated for 6 h in the absence (CTRL) and in the presence of of gibbilimbol B/eriopodol A (30 µg/mL) or erioquinol (10 µg/mL). Scale bars = 50 µm (bright field) and 10 µm (DAPI). Lower panels represent enlarged image details. (**C**) Quantitative analysis of tetramethylrhodamine methyl ester (TMRM) fluorescence changes over time in MCF7 (left panel) and U373 (righ panel) cells in the absence (CTRL) and in the presence of gibbilimbol B/eriopodol A (30 µg/mL) or erioquinol (10 µg/mL). Results are expressed by setting TMRM fluorescence in the respective control (vehicle-treated) samples, i.e., absence of compounds, as 1. *** *p* < 0.0001 relative to CTRL. Images and data are representative of four independent experiments.

In addition, cells treated with 30 µg/mL gibbilimbol B and eriopodol A displayed activation of caspase 9 and 7 at 3 h and 6 h, as showed by western blot analysis (Figure 6A,B). On the other hand, erioquinol (10 µg/mL) treated cells did not display any sign of caspase 7 activity even at later time point (Figure 6C). These results were confirmed by immunofluorescence experiments. Indeed, a time- dependent and intensive cleaved-caspase 7 staining was detected in the cytoplasm of MCF7 cells in the presence of gibbilimbol B and eriopodol A while positive cells were absent following the administration of erioquinol (Figure 6D). The activation of caspase 7 by 6 h gibbilimbol B and eriopodol A (30 µg/mL) but not erioquinol (10 µg/mL) was achieved also in U373 cells (Figure 7A). The fact that these cells displayed apoptotic and non-apoptotic features in the presence of gibbilimbol B/eriopodol A and erioquinol, respectively (Figure 7B), similarly to what obtained in MCF7 cells, indicate that cell death mechanisms of the compounds are comparable among cell lines. Accordingly, the activation of caspase 7 by gibbilimbol B and eriopodol A but not erioquinol was observed also in MCF10 cells (Figure S6A). 

In order to better describe the mechanism behind the activity of the compounds, we investigated mitochondria functionality with tetramethylrhodamine methyl ester (TMRM), a red fluorescent dye that is sequestered by active mitochondria. Of note, MCF7 and U373 cells treated for increasing time with 30 µg/mL gibbilimbol B/eriopodol A or 10 µg/mL erioquinol presented a comparable decrease in TMRM fluorescence vs. control, with MCF7 cells full responding within 1 h (Figure 7C). This indicates low mitochondria membrane potential likely associated to the destabilisation of the mitochondrial membrane systems. 

**Figure 8:**
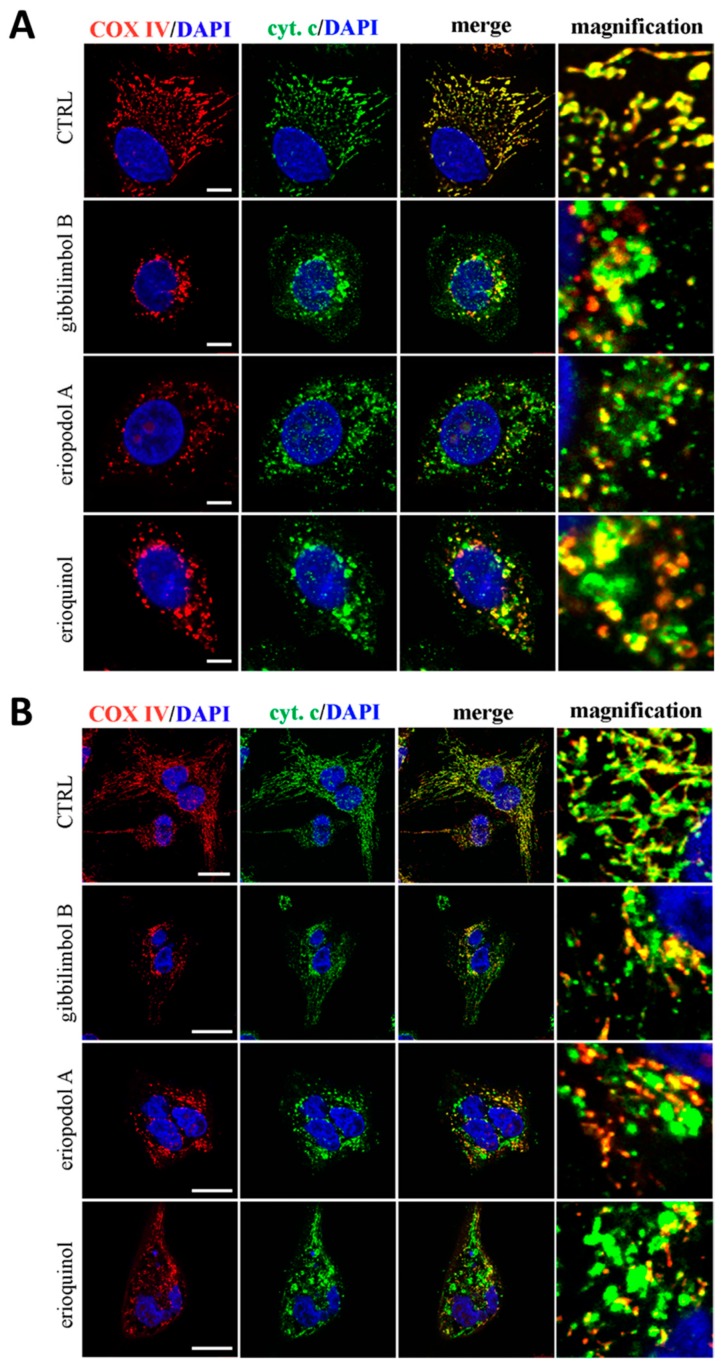
Confocal microscopy for co-localisation of cytochrome c with mitochondria. (**A**) MCF7 and (**B**) U373 cells were treated for 3 h in the absence (CTRL, control) and in the presence of gibbilimbol B/eriopodol A (30 µg/mL) or erioquinol (10 µg/mL). Cells were then stained for cytochrome c (green) and the mitochondrial marker COX IV). 4’,6-diamidine-2’-phenylindole dihydrochloride (DAPI) (blue) was used for nuclei detection. The images are representative of three independent experiments. Scale bars: 10 µm (MCF7) and 25 µm (U373). Panels on the right represent enlarged image details.

The fact that the three compounds similarly induce mitochondria membrane permeabilisation both in MCF7 and U373 cells, was further confirmed by the subcellular location of cytochrome c. As shown in Figure 8A,B, 3 h administration of gibbilimbol B/eriopodol/erioquinol induced an alteration in the cytochrome c staining pattern from mitochondrial (co-localisation with COX IV, a marker for mitochondria), to a more cytosolic distribution (presence of many clusters which did not overlap with COX IV), indicating a release of cytochrome c from the dysfunctional mitochondria. 

**Figure 9:**
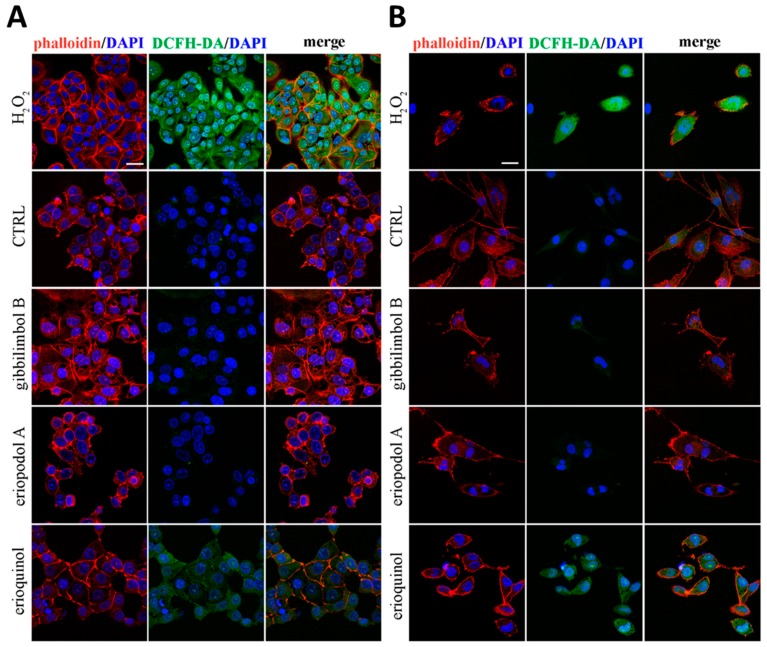
Confocal microscopy for reactive oxygen species (ROS) detection. (**A**) MCF7 and (**B**) U373 cells were treated for 6 h in the absence (CTRL, control) and in the presence of gibbilimbol B/eriopodol A (30 µg/mL) or erioquinol (10 µg/mL). Cells were then stained for ROS (2’-7’dichlorofluorescin diacetate - DCFH-DA, green). 4’,6-diamidine-2’-phenylindole dihydrochloride (DAPI) (blue) and phalloidin (red) were used for nuclei and cytoskeleton detection, respectively. The images are representative of three independent experiments. Scale bar: 25 µm.

Damaged mitochondria are considered as the main source of reactive oxygen species (ROS) which play major roles in the fate of cancer cells [63]. Noteworthily, MCF7 and U373 cells staining with 2’-7’dichlorofluorescin diacetate (DCFH-DA), a permeant fluorogenic dye cell reagent that measures hydroxyl, peroxyl and other ROS activity, revealed that erioquinol effect (10 µg/mL, 6 h) is characterised by marked accumulation of ROS, which are absent in cells treated with gibbilimbol B and eriopodol A (30 µg/mL, 6 h) (Figure 9A,B). Together with lack of caspase activation, aberrant ROS production is another divergence between gibbilimbol B/eriopodol A and erioquinol-induced cell death. In this respect, erioquinol is likely inducing a robust mitochondrial stress which results in ROS production and release into the cytoplasm.

**Figure 10:**
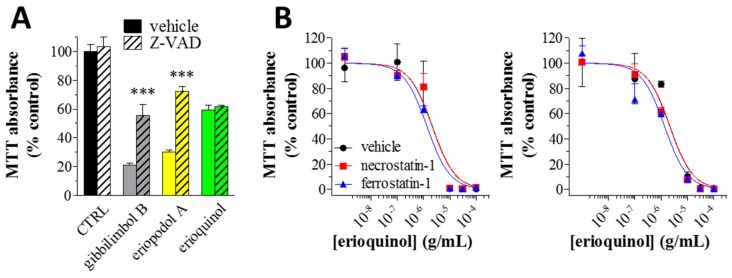
*Piper* genus-derived compounds induce caspase-dependent and independent loss of cell viability. (**A**) MCF7 cells were cultured in the absence (CTRL, control) and in the presence of 30 μg/mL gibbilimbol B/eriopodol A or 10 µg/mL erioquinol for 6 h, before 3-(4,5-dimethylthiazol-2-yl)-2,5-diphenyltetrazolium bromide (MTT) assay. The pan-caspase inhibitor Z-VAD-(OMe)-FMK (50 μM) or its vehicle were used as well. Results are expressed by setting the absorbance of the reduced MTT in the CTRL, as 100%. Data are representative of four-twelve independent experiments. *** *p* < 0.0001 relative to the respective compound alone, i.e., + Z-VAD vehicle. (**B**) MCF7 (left panel) and U373 (right panel) cells were treated with increasing concentrations of erioquinol for 24 h before MTT assay. Erioquinol was administered both in the absence (vehicle) or in the presence of 50 μM necrostatin-1 and 10 μM ferrostatin-1 (2 h pre-treatment), a necroptosis and ferroptosis inhibitor, respectively. Results are expressed by setting the absorbance of the reduced MTT in the control samples (absence of erioquinol) as 100%. The data points are representative of four independent experiments.

Finally we confirmed as apoptotic the effect of gibbilimbol B and eriopodol A by inhibiting their cytotoxic activity with the pan-caspase inhibitor Z-VAD-(OMe)-FMK. As displayed by MTT assays (Figure 10A), the loss of cell viability in MCF7 cells treated with 30 µg/mL gibbilimbol B and eriopodol A was significantly inhibited when 50 µM Z-VAD-(OMe)-FMK was simultaneously added to the 6 h treatment protocol, demonstrating the dependency on caspases of the two compounds. However, the simultaneous addition of Z-VAD-(OMe)-FMK did not affect the activity of 10 µg/mL erioquinol. Taken together our data data demonstrate that gibbilimbol B and eriopodol A effectively induced intrinsic apoptosis triggered by mitochondrial membrane permeabilisation, release of cytochrome c, an early induction of initiator caspase 9, and a consecutive activation of effector caspase 7. Erioquinol, although it affects comparably mitochondrial functions, appears to act in a different manner, i.e., involving mitochondrial ROS release and non-apoptotic/caspase-independent mechanisms. Caspase-independent cell death was first described to affect mitochondria potential, and eventually mitochondrial outer membrane permeabilisation [64], although not followed by caspase activation. Those features resemble the outcome of erioquinol treatment.

Several forms of regulated cell death manifest with a morphology different from apoptosis [65,66], and many compounds from nature source can induce non-apoptotic programmed cell death in cancer cells [67]. Among them, necroptosis can be partially rescued by the receptor-interacting serine-threonine kinase 1 inhibitor necrostatin-1 and ferroptosis by ferrostatin-1, an inhibitor of lipid peroxidation. We thus treated MCF7 and U373 cells with increasing concentrations of erioquinol (24 h) with or without 50 µM necrostatin-1 and 10 µM ferrostatin-1 (2 h pre-treatment). As shown in Figure 10B, the concentration-dependent inhibition of MTT absorbance did not change, suggesting erioquinol-induced death was independent from necroptosis and ferroptosis, two cell death pathways known to be caspase-independent [65,66]. ROS were recently linked to a caspase-independent form of cell death, which cannot be rescue by necrostatin-1 or ferrostatin-1 treatment, and therefore not imputable to either necroptosis or ferroptosis [68]. Treatment with erioquinol might lead to a similar cascade of events, although additional work is required to fully characterise the role of ROS and the cell death process induced by this *Piper* genus-derived compound.

### 2.4. XIAP as a Molecular Target of Piper Genus-Derived Compounds

XIAP-mediated inhibition of apoptosis goes through its reversible binding to active caspase-9, via its BIR3 domain, and caspase-3/7 when stabilised to XIAP-BIR2 domain [69,70,71]. It has been also demonstrated that XIAP controls different pathways functionally uncoupled to caspases, leading to the possibility that XIAP system might control cell death/survival through multiple mechanisms [32,34,72,73,74,75,76,77,78,79,80,81,82].

**Figure 11:**
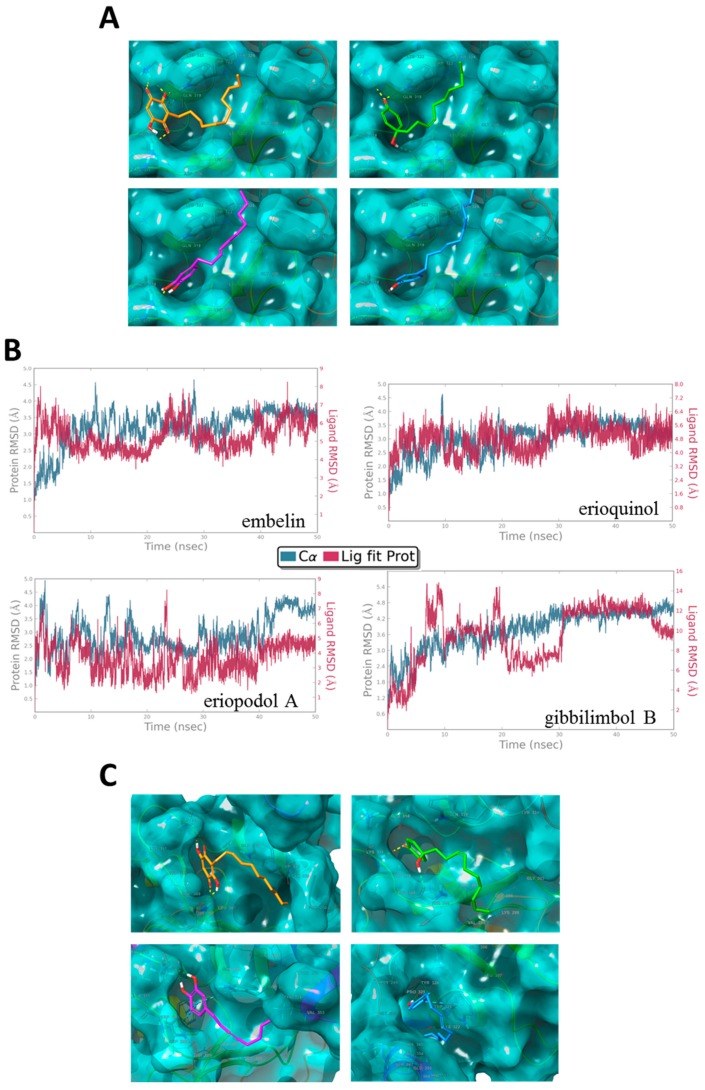
X-linked inhibitor of apoptosis protein (XIAP) as a molecular target of *Piper* genus-derived compounds. (**A**) Molecular docking and (**C**) dynamics analysis of embelin (orange), erioquinol (green), eriopodol A (purple) and gibbilimbol B (blue) in complex with the baculovirus IAP repeat (BIR)-3 domain of XIAP (PDB code 5C83). Interacting residues are displayed in wireframe, hydrogen bonds are displayed in yellow dot lines and π-π stacking interactions are displayed in blue dot lines. (**B**) Protein-ligand root mean square deviation (RMSD) trajectory of the atomic positions for ligands (red, Lig fit Prot) and the receptor (blu, C⍺ positions) BIR-3 domain of XIAP, for the dynamics trajectory of 50 ns.

Embelin, a natural benzoquinone with potential therapeutic interest, has been isolated from the fruit of the *Embelia* ribes and discovered through molecular docking analysis of over 8200 molecules as a potent small molecule XIAP inhibitor that binds to the XIAP-BIR3 domain [83,84,85,86]. It should be noted that embelin displays chemical features similar to those of erioquinol, eriopodol A, and gibbilimbol B [83]. We assessed if erioquinol, eriopodol A, and gibbilimbol B are able to bind to the XIAP-BIR3 domain in a similar way of embelin. Using molecular docking analysis and molecular dynamics simulations for embelin and isolated new compounds, it was found the structural basis of the predicted interactions with the BIR3 domain of XIAP. Figure 11A provides a general view of the docked conformations obtained for gibbilimbol B, eriopodol A, erioquinol, and embelin. Interestingly, the binding site for gibbilimbol B, eriopodol A, and erioquinol is the same binding site of embelin and with similar energy and binding mode. All docked compounds fits in to the P1, P2 and P3 of the P1–P4 pockets reported for the binding site of the XIAP-BIR3 domain in complex with Smac, the endogenous antagonist ligand of IAPs [35,87,88]. 

Experimental structures of the XIAP-BIR3 domain in different complexes with embelin, Smac or Smac mimetics and non-peptidomimetics small molecules, revealed that residues GLY306, THR308, GLU314, TRP323 and TYR324 are crucial residues involved in the interaction with the BIR3 domain of XIAP [35,41,86,89]. The results of the docking experiments show a possible binding mode for gibbilimbol B, eriopodol A, and erioquinol. Accordingly, the phenolic ring of gibbilimbol B and eriopodol A forms hydrogen bonds with LYS311 and GLU314 (Figure 11A), the quinol ring of erioquinol forms three hydrogen bonds with THR308, LYS322, and TRP323, while residues GLY306, LEU307, TRP323, and TYR324 of the XIAP-BIR3 domain forms hydrophobic interactions with the tail of the alkenyl derivatives. 

In addition, molecular dynamics simulations for 50 ns were carried out to assess the stability of the protein-ligand complexes between the docked compounds and the BIR3 domain of XIAP. The stability of the modelled complex of alkenyl derivatives and embelin was confirmed during the period of simulation by little variations in the root mean square deviation (RMSD) trajectory (Figure 11B). Although some changes were observed in the interacting residues of XIAP BIR-3 domain after molecular dynamics simulations (Figure S7), the preferred location of the binding mode for all evaluated ligands were maintained in the pockets P1-P4 of BIR-3 domain of XIAP during the period of simulation (Figure 11C). Also, the binding mode obtained in the docking and dynamics simulations for embelin are according to the interactions pattern determined experimentally by NMR studies in the XIAP-embelin complex, which revealed that TRP323 of the BIR3 domain of XIAP are crucial in the binding of embelin [86]. These findings strongly suggest the highly stable complex formation between the BIR-3 domain of XIAP and the alkenyl derivatives.

**Figure 12:**
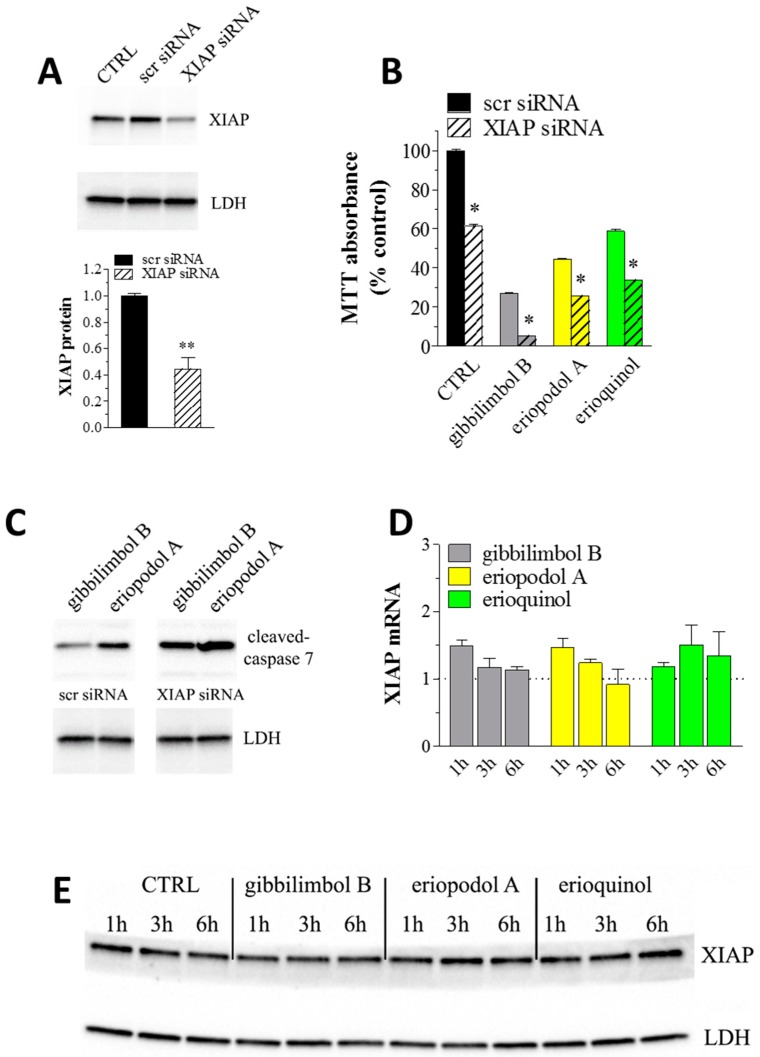
X-linked inhibitor of apoptosis protein (XIAP) as a molecular target of *Piper* genus-derived compounds. (**A**) Western blot analysis of XIAP in MCF7 cells both untransfected (CTRL, control) or transfected for 24 h with a XIAP-specific and scrambled targeting (scr) siRNA (50 nM). Lactate dehydrogenase (LDH) was used as internal standard. Low panel: densitometric analysis expressed as fold change of scr siRNA. Images and data are representative of three independent experiments. **P < 0.001 relative to scr siRNA. (**B**) MCF7 cells were transfected for 24 h with a XIAP-specific or scr siRNA (50 nM) and then cultured in the absence (CTRL) and in the presence of 30 μg/mL gibbilimbol B/eriopodol A or 10 µg/mL erioquinol for 6 h, before 3-(4,5-dimethylthiazol-2-yl)-2,5-diphenyltetrazolium bromide (MTT) assay. Results are expressed by setting the absorbance of the reduced MTT in the scr siRNA CTRL, as 100%. Data are representative of three independent experiments. * *p* < 0.0001 relative to the respective scr siRNA. (**C**) Western blot analysis of cleaved-caspase 7 in MCF7 cells transfected for 24 h with a XIAP-specific or scr siRNA (50 nM) and then cultured in the presence of 30 μg/mL gibbilimbol B and eriopodol A for 6 h. LDH was used as internal standard. Images are representative of three independent experiments. (**D**) Real-time PCR and (**E**) Western blot analysis of XIAP mRNA and protein expression, respectively, in MCF7 cells treated for increasing times in the absence (CTRL) and in the presence of 30 μg/mL gibbilimbol B/eriopodol A or 10 µg/mL erioquinol. β-actin (PCR) and LDH (Western blot) were used as internal standards. PCR results are expressed as fold change of respective CTRL, set as 1. Images and data are representative of three independent experiments.

XIAP is highly expressed in different human tumour cells and cancer specimens from patients and plays an important role in conferring chemoresistance [33,90]. Because XIAP blocks apoptosis at the downstream effector phase, where multiple signalling events may converge, it represents an attractive molecular target for the design of new anti-cancer drugs [32,33,34,35,36,37,38,39,40,41,43]. Two broad approaches have been taken to develop clinical inhibitors of XIAP—antisense oligonucleotides, targeting the entire protein, and small molecule inhibitors, binding a single domain. Small molecule inhibitors offer the potential of more rapid inhibition of their target in vivo and more predictable duration of action [34,41]. Among the small molecule phytochemicals, the XIAP inhibitor embelin exhibited cytotoxic activity in various human tumoural cells, including breast cancer [83,84,85,86,91]. In addition, the withaferin-A induced cytotoxicity in human breast cancer cells was associated with suppression of XIAP protein [92] and berberine was shown to induce apoptosis in tumours, likely through the inhibition of XIAP [93]. The just mentioned molecular modelling of our new molecules binding to XIAP-BIR3 domain drove us to examine if they shared a similar activity with already described XIAP inhibitors. With the aim of understanding the role of XIAP in the cell death phenotype, we first determined if our cellular model is anyhow affected by XIAP depletion. Using the Lipofectamine reagent, MCF7 cells were transiently transfected with a XIAP-specific or a scrambled targeting siRNA. When treated with 50 nM of siRNA for 24 h, the protein levels of XIAP markedly decreased to ca. 45% compared to control siRNA transfected samples (Figure 12A) indicating a partial depletion of XIAP. In agreement with previous indications [94,95,96], the outcome in viability of XIAP knockdown in MCF7 cells, which showed a significant reduction (ca. 40%) in MTT absorbance upon depletion of XIAP (Figure 12B), led us to the conclusion that MCF7 cells depend on XIAP for survival since death mechanisms are neutralised by physiological levels of XIAP. We then tried to add clues on the involvement of XIAP in the cytotoxic effect of *Piper* genus-derived compounds. As shown in Figure 12B, XIAP downregulation in MCF7 cells significantly enhanced the toxicity, as measured by MTT absorbance, of 6 h administration of gibbilimbol B (30 μg/mL), eriopodol A (30 μg/mL) and erioquinol (10 μg/mL) indicating their combined action with XIAP siRNA in inhibiting cell viability. Since 100% knockdown was never achieved with siRNA technique (the absence of detectable XIAP after siRNA transfection, i.e., by XIAP siRNA at 100 nM for 24 h, paralleled the increase of cleaved-caspase 7 levels and the complete loss of MCF7 cell viability (Figure S6D and data not shown)), it is reasonable to assume that that the effects of gibbilimbol B/eriopodol A/erioquinol on the residual XIAP protein in the siRNA-treated cells further induced MCF7 cell death. On the other hand, similar results (additive effect) would be achieved if the compounds target cytotoxic pathways other than XIAP. However, although this is not a formal biological evidence, the simplest explanation of the combined action is a XIAP-mediated mechanism accounting for, at least in part, the cytotoxicity of our new compounds. Accordingly, the positive effects of gibbilimbol B and eriopodol A on caspase 7 activity robustly increased after XIAP silencing (Figure 12C). Downregulation of XIAP by siRNA is known to sensitise human breast cancer cells to death mediated by different chemical agents [94,97]. Finally, using real-time PCR and western blot assays to measure XIAP expression, we found that cell exposure to gibbilimbol B, eriopodol A, and erioquinol at increasing times did not significantly modify mRNA (Figure 12D) and protein levels (Figure 12E) of XIAP. Overall, our data exclude a role of gibbilimbol B/eriopodol A/erioquinol on the regulation of XIAP expression but rather are consistent with the antagonism of XIAP activity through their binding to XIAP-BIR3 domain.

Since escape from apoptosis is one of the preeminent features of cancer, pharmacological interest in targeting endogenous apoptosis inhibitors, such as B-cell lymphoma (BCL)-2 and IAPs family members, has been constant [32,33,34,35,36,37,38,39,40,41,42,43,82,98,99]. The efforts, including clinical trials, directed towards identifying small molecules inhibitors of the BCL-2 family of proteins and promote apoptosis with the so-called BH3 mimetics, that mimic the action of certain BH3-only proteins [98], proved the releasing of “apoptosis brakes” as a winning strategy to induce primary cell death in cancer or to sensitise tumour to chemotherapy. Differently to BCL-2 family members, IAPs, and in particular XIAP, have a late role in the apoptotic timeline, and they target already active caspases to prevent cell death. The structural data surrounding the interaction between the BIR3 domain of XIAP and caspases suggest that small molecules that bind the BIR3 pocket of XIAP could mimic the action of Smac and inhibit the interaction between XIAP and caspase [34,41]. Interestingly its multi-domain structure makes XIAP a component of multiple cellular pathways, not only the ones leading to apoptosis. XIAP versatility has been highlighted in inflammation and inflammatory cell death, such as necroptosis [32,79,80,81,82]. Even though these aspects are yet to be completely elucidated, we suggest here - in addition to the widely described activity of XIAP inhibitors in apoptosis induction (gibbilimbol B and eriopodol A) —an interesting example of how the pharmacological targeting of XIAP-BIR3 domain can go beyond the simple induction of apoptosis—and extends its influence in modulating cell death signalling events other than caspase-activation (erioquinol). The relevance of non-apoptotic cell death in cancer treatment has recently gained interest as a means to simultaneously targetting tumours and enhancing the inflammatory response [100]; XIAP, in this context, is an interesting crossroad of pathways involved in both cell death and inflammation.

## 3. Materials and Methods

### 3.1. Extraction and Isolation of Natural Compounds

*P. eriopodon* was collected in Fusagasuga, in the Department of Cundinamarca (Colombia). The plant material was identified by Dr. Adolfo Jara Muñoz at Herbario Nacional Colombiano and a voucher specimen (COL516757) was deposited at the Instituto de Ciencias Naturales, Universidad Nacional de Colombia. 

Dried and powdered leaves of *P. eriopodon* (1.14 Kg) were extracted exhaustively with ethanol 96% (3 × 5L) at room temperature. After filtration, the solvent was evaporated under reduced pressure, to yield 103.6 g of crude extract. The crude extract (100.0 g) was subjected to silica gel flash chromatography and eluted with a step gradient of toluene/ethyl acetate (0:100, 20:80, 40:60, 60:40, 80:20 and 0:100 (V/V)) to afford eight fractions. Fraction 1 (34.2 g) was chromatographed over silica gel, eluting with a mixture of a three-phase *n*-hexane/dichloromethane/ethyl acetate (25:70:5) solvent system to afford ten fractions (A to J). Fraction E (10.0 g) was chromatographed over Sephadex LH-20 (4.5 × 45 cm, *n*-hexane/chloroform/methanol, 2:2:1) to give six fractions (E1 to E6). In agreement with a previous report [29], compound **1** (7.93 g) was obtained from fraction E3, after column chromatography on Sephadex LH-20 (4.5 × 30 cm, *n*-hexane/acetone/methanol, 2:2:1). Fraction E4 (974.6 mg) was submitted to column chromatography on Sephadex LH-20 (4.0 × 20 cm, *n*-hexane/acetone/methanol, 2:2:1) to yield six fractions (E4.1 to E4.6). Compound **3** (33.2 mg) was obtained from fraction E4.4 (378.8 mg) through Sephadex LH-20 (2.0 × 25 cm, *n*-hexane/acetone/methanol, 2:2:1) and silica gel column chromatography eluted with *n*-hexane/acetone 8:2. Fraction E5 (2.19 g) was subjected to column chromatography on silica gel using a mixture of toluene/ethyl acetate (9:1) to afford ten fractions (E5.1 to E5.10). Fraction E5.3 (153.2 mg) was purified by flash chromatography to yield compound 4 (20.0 mg).

Fraction 2 (8.0 g) was submitted to silica gel column chromatography eluted with *n*-hexane/ethyl acetate 8:2, yielding seven fractions (K–Q). Fraction Q was subjected to flash chromatography eluted with dichloromethane/acetone (7:3) to yield six fractions (Q1–Q6). Fraction Q3 (1.44 g) was subjected to column chromatography over Sephadex LH-20 (4.0 x 20 cm, hexane/acetone/methanol, 2:2:1) to afford six fractions (Q3.1 to Q3.6). Fraction Q3.4 (239.4 mg) was chromatographed over Sephadex LH-20 (4.0 × 20 cm, hexane/chloroform/methanol, 2:2:1) and then purified by silica gel column chromatography eluted with *n*-hexane/acetone (7:3) to yield compound **5** (4.0 mg). Fraction Q6 (570.5 mg) was subjected to flash chromatography eluted with *n*-hexane/acetone 7:3 to afford seven fractions (Q6.1–Q6.7). Compound **2** (166.0 mg) was obtained from fraction Q6.1.

### 3.2. General Chemical Methods

Flash chromatography was carried out with silica gel (230–400 mesh; Merck, Darmastadt, Germany), column chromatography was performed using silica gel (70–230 mesh; Merck) and Shepadex^®^ LH20 (Sigma-Aldrich, St. Louis, MO, USA), analytical thin layer chromatography was performed using precoated silica gel plates 60 F_254_ (0.25 mm, Merck). ^1^H and ^13^C NMR 1D and 2D (COSY, HMQC and HMBC) spectra, were recorded on an Avance 400 spectrometer (Bruker, Millerica, MA, USA) at 400 MHz for ^1^H and 100 MHz for ^13^C using the solvent peaks as internal references, the spectra were recorded in CDCl_3_ and MeOD (Merck). High-resolution mass data were collected on an Accurate-Mass quadrupole Time-of-Flight (q-TOF) (Agilent Technologies, Santa Clara, CA, USA) mass spectrometer, ESI negative mode, Nebuliser 50 (psi), Gas Flow 10 L/min, Gas Temp 350 °C. Fragmentor 175 V, Skimmer 75 V, Vpp 750 V.

### 3.3. Cell Culture and Chemicals

Human U373 glioma, MCF7 breast cancer, A549 lung cancer and PC-3 prostate cancer cells were grown in Dulbecco’s Modified Eagle Medium (DMEM), supplemented with 10% foetal bovine serum, 2 mM glutamine, 100 U/mL penicillin/streptomycin, at 37 °C in a humidified atmosphere containing 5% CO_2_ (logarithmic growth phase, routine passages every 3 days). The human breast epithelial cell line MCF10 was cultured in DMEM/F12 Ham’s Mixture supplemented with 5% horse serum, epithelial growth factor 20 ng/mL, insulin 10 μg/mL, hydrocortisone 0.5 mg/mL, cholera toxin 100 ng/mL, and 100 U/mL penicillin/streptomycin. HUVEC were grown in EGM-2 Endothelial Cell Growth Medium-2 BulletKit (Lonza, Basel, Switzerland), according to the manufacturer’s protocol. 

Foetal bovine serum, horse serum, glutamine and penicillin/streptomycin were obtained from Euroclone (Milano, Italy). TMRM was purchased from ThermoFisher Scientific (Waltham, MA, USA) while necrostatin-1 and ferrostatin-1 were obtained from Santa Cruz Biotechnology (Dallas, TX, USA). Where not indicated, the reagents were purchased from Sigma-Aldrich.

### 3.4. MTT Assay

U373, MCF7, A549, PC-3, HUVEC, and MCF10 cell viability was determined by MTT assay using published protocols [101,102,103,104,105]. MTT absorbance was quantified spectrophotometrically using a Glomax Multi Detection System microplate reader (Promega, Milano, Italy).

### 3.5. TUNEL Assay

Using published protocols [106,107], MCF7 or U373 cells cultured in 120-mm coverslips were fixed in 4% paraformaldehyde in 0.1 M phosphate buffer (PB), pH 7.4, for 10 min. The TUNEL method (DeadEnd Fluorometric TUNEL System, Promega) was used to assay apoptosis, according to the manufacturer’s protocol. DAPI (nuclei detection) staining was also performed.

### 3.6. Immunofluorescence Microscopy Analysis

Using published protocols [106,108], MCF7 or U373 cells cultured in 120-mm coverslips were fixed in 4% paraformaldehyde in 0.1 M PB, pH 7.4, for 10 min. Cells were pre-incubated for 1 h min with 5% of normal goat serum (Life Technologies, Monza, Italy) in 0.1 M PB (pH 7.4) containing 0.1% Triton X-100, before overnight incubation with the rabbit monoclonal anti-cleaved caspase 7 (Cell Signaling Technology, Danvers, MA, USA). In double-label immunofluorescence experiments, the mouse monoclonal anti-cytochrome c primary antibody (Cell Signaling Technology) was used in conjunction with the rabbit monoclonal primary antibody directed to COX IV (Cell Signaling Technology). For fluorescence detection, coverslips were stained with the appropriate Alexa Fluor secondary antibodies (Life Technologies) and mounted on glass slides in a ProLong Gold Antifade Mountant (Life Technologies). DAPI and/or fluorescein phalloidin (cytoskeleton detection) staining was also used. Cells were analysed with a DMI4000 B automated inverted microscope equipped with a DCF310 digital camera (Leica Microsystems, Wetzlar, Germany). When indicated, confocal imaging was performed with a TCS SP8 System (Leica Microsystems). Image acquisitions were controlled by the Leica Application Suite X.

### 3.7. Annexin V Staining 

MCF7 cells were incubated with 5 μg/mL Annexin V-fluorescein isothiocyanate (FITC) to assess the phosphatidylserine exposure on the outer leaflet of the plasma membrane, and 5 μg/mL PI (DNA-binding probe) to exclude necrotic cells in binding buffer (10 mM HEPES, 140 mM NaCl, 2.5 mM CaCl_2_) [109]. Cell staining was analysed by Gallios Flow Cytometer (Beckman-Coulter, Brea, CA, USA) and the software FCS Express 4 (De Novo System, Portland, OR, USA).

### 3.8. Western Blotting

Using published protocols [107,110,111], MCF7 and MCF10 cells were homogenised in RIPA lysis buffer, supplemented with a cocktail of protease inhibitors (cOmplete; Roche Diagnostics, Milano, Italy). Equal amounts of proteins were separated by 4–20% SDS-polyacrylamide gel electrophoresis (Criterion TGX Stain-free precast gels and Criterion Cell system; Bio-Rad, Hercules, CA, USA) and transferred onto nitrocellulose membrane using a Bio-Rad Trans-Blot Turbo System. When indicated, the membranes were probed using the rabbit monoclonal anti-cleaved caspase 7 and anti-XIAP (Cell Signaling Technology) primary antibodies. After the incubation with the appropriate horseradish-peroxidase-conjugated secondary antibody (Cell Signaling Technology), bands were visualised using the Clarity Western ECL substrate with a ChemiDoc MP imaging system (Bio-Rad). To monitor for potential artefacts in loading and transfer among samples in different lanes, the blots were routinely treated with the Restore Western Blot Stripping Buffer (ThermoFisher Scientific) and re-probed with the goat anti-Lactate dehydrogenase (LDH)-A (Santa Cruz Biotechnology) and the mouse anti-vinculin primary antibodies. The stain-free gel was used as loading control as well. When appropriated, bands were quantified for densitometry using the Bio-Rad Image Lab software.

### 3.9. Mitochondrial Membrane Potential Analysis

Using published protocols [112], mitochondria of MCF7 and U373 cells were labeled using TMRM, a voltage-sensitive cationic lipophilic dye, partitioning and accumulating in the mitochondrial matrix based upon the Nernst equation. After treatments, cells were trypsinised, counted and incubated with 100 nM TMRM for 30 min at 37 °C. Fluorescence was measured by using a Glomax Multi Detection System microplate reader (Promega), excitation wavelength: 525 nm; emission wavelength: 580–640 nm). After background subtraction, the data were normalised on cell number.

### 3.10. Measurement of ROS

MCF7 or U373 cells cultured in 120-mm coverslips were exposed to 30 µM DCFH-DA (0.1 M PB, pH 7.4) and fixed in 4% paraformaldehyde for 20 min. For fluorescence detection, coverslips were mounted on glass slides and observed with a laser-scanning confocal microscope (TCS SP8 System and Application suite X, Leica Microsystems). DAPI and fluorescein phalloidin (nuclei and cytoskeleton detection, respectively) staining was also used. 

### 3.11. Molecular Modeling

AutoDock4 was used to carry out the molecular docking. The Protein Data Bank crystallographic structure PDB 5C83 was considered as receptor model [87]. The preparation of the macromolecule was made with PyMOL (version 2.0, PyMol Molecular Graphics, Schrodinger, New York, NY, USA) System) and XIAP-BIR3 domain was selected as receptor [113]. Energy maps was established with Autogrid4 involving all atom types. After 25 million of energy evaluations in the binding pocket and using a grid of 50 × 50 × 50 points, all conformations of the ligand were clustered according to the energy and conformations. The docking results were visualised using the computational program Maestro 11.6. The molecular dynamics simulations were carried out with Desmond simulation package of Maestro (Desmond Molecular Dynamics System; D. E. Shaw Research, New York, NY, USA, 2016) using the OPLS 2005 force field parameters. A solvated system (TIP3P) and a predefined model for electrically neutral system (physiological concentrations of monovalent ions, NaCl 0.15 M) were used in an orthorhombic box and maintained at constant temperature of 300 K for all simulations. The dynamics simulations were analysed using the Simulation Interaction Diagram tool of Desmond package, monitoring the behaviour and stability of simulations by RMSD of the ligand and protein atom positions in time.

### 3.12. RNA Interference

Gene silencing of XIAP in MCF7 cells was performed as previously published [106]. Briefly, according to the manufacturer’s protocol, iBONI siRNA Pool (Riboxx, Radebeul, Germany) targeting human XIAP were mixed to Lipofectamine RNAiMax transfection reagent (Life Technologies). iBONI siRNA Pool negative control (Riboxx) (scrambled targeting siRNAs) was also used. The mix was added to cultured MCF7 cells at a siRNA concentration of 50 nM for 24 h. 

### 3.13. Real-Time PCR

The analysis of mRNA expression was performed as previously described [106,114,115]. Briefly, total RNA from MCF7 cells was extracted with the High Pure RNA Isolation Kit (Roche Applied Science, Mannheim, Germany), according to the manufacturer’s protocol. First-strand cDNA was generated from 1 μg of total RNA using iScript Reverse Transcription Supermix (Bio-Rad). Primer pairs (Eurofins Genomics, Milano, Italy) for XIAP (NM_001167; forward ACCGTGCGGTGCTTTAGTT, reverse TGCGTGGCACTATTTTCAAGATA) and β-actin (NM_001101; forward ATAGCACAGCCTGGATAGCAACGTAC, reverse CACCTTCTACAAT GAGCTGCGTGTG) were designed to hybridise to unique regions of the appropriate gene sequence. PCR was performed using SsoAdvanced Universal SYBR Green Supermix and the CFX96 Touch Real-Time PCR Detection System (Bio-Rad). The fold change was determined relative to the selected control sample after normalising to β-actin (internal standard) by the formula 2^−ΔΔCT^.

### 3.14. Statistics

Statistical significance of raw data between the groups in each experiment was evaluated using unpaired Student’s *t*-test (single comparisons) or one-way ANOVA followed by the Newman-Keuls post-test (multiple comparisons). The IC_50_ and E_max_ concentration were determined by non-linear regression curve analysis of the concentration-effect responses. Potency values among concentration-response curves were compared with the F-test. Data belonging from different experiments were represented and averaged in the same graph. The GraphPad Prism software package (GraphPad Software, San Diego, CA, USA) was used. The results were expressed as means ± standard error of mean (SEM) of the indicated n values.

## 4. Conclusions

**Figure 13:**
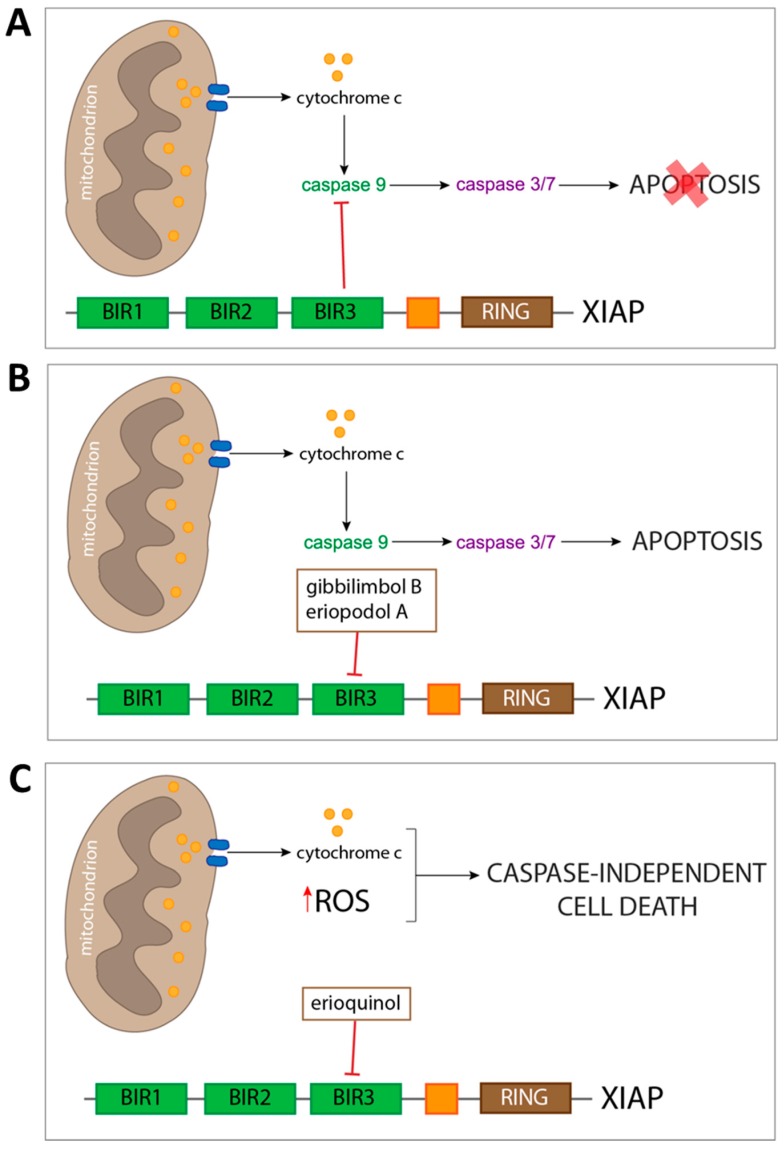
Schematic picture depicting cell death mechanisms of *Piper* genus-derived compounds. Escape of both intrinsic and extrinsic apoptosis is a common feature of cancer cells. (**A**) This hallmark is often carried out by overexpressing anti-apoptotic proteins, such as X-linked inhibitor of apoptosis protein (XIAP), which prevents the execution of apoptosis by binding of its baculovirus IAP repeat (BIR) 3 domain to already active initiator caspase 9. In order to counteract this resistance to cell death, several cancer pharmacological therapies have the aim of removing the ‘molecular brakes’ to apoptosis sensitising cancer cell to undergo loss of viability. The approach we described includes the use of three compounds from *Piper* genus plants which were predicted to bind XIAP-BIR3 domain. (**B**) Two of the compounds (gibbilimbol B and eriopodol A) were shown to induce a classical pro-apoptotic response, including mitochondrial outer membrane polarisation, release of cytochrome c, and subsequent activation of both initiator and effector caspases. (**C**) Despites triggering a similar response at the mitochondria level, erioquinol does not act through the apoptotic machinery, and results in a caspase-independent cell death characterised by cytoplasmic reactive oxygen species (ROS) accumulation.

This study adds to the renewed biological interest in natural derived compounds, by presenting a chemical and biological characterisation of new small organic molecules derived from *Piper* genus plants. Following a recent preliminary report of gibbilimbol B as cytotoxic in breast cancer cell lines, we explored this observation by comparing it to similarly structured new molecules. Erioquinol that appeared to be the most potent compound versus gibbilimbol B and eriopodol A was listed as an intermediate. A more detailed investigation of the biological mechanism behind these molecules’ activity in shaping cell viability revealed induction of caspase-dependent apoptosis following exposure of tumour cells to gibbilimbol B and eriopodol A and, interestingly, display of caspase-independent/non-apoptotic features in cell treated with erioquinol. In silico modelling and molecular approaches gave us a first preliminary insight into the molecular target of *Piper* genus compounds, the anti-apoptotic protein XIAP (Figure 13). Of note, an already identified XIAP inhibitor shared structural and binding similarities with them. The appeal of XIAP as a therapeutic target in cancer is not restricted to inhibition of apoptosis, but comprehends the regulation of other cellular physiological aspects, such as control of caspase-independent cell death. The molecular signature behind our observation opens important implications to further dissect the role of XIAP and for the development of novel XIAP antagonists for cancer treatment.

## Figures and Tables

**Table 1:** Inhibitory effects of *Piper* genus-derived compounds on human cancer cell viability.

Compound	IC_50_ (μg/mL)	
	U373 Cells	MCF7 Cells
**Gibbilimbol B**	16.79	16.44
**Eriopodol A**	11.12	10.12
**Eriopodol B**	31.91	29.36
**Eriopodol C**	14.30	16.30
**Erioquinol**	1.78	2.63

3-(4,5-dimethylthiazol-2-yl)-2,5-diphenyltetrazolium bromide (MTT) assay was performed treating cells for 24 h in the absence (vehicle) or in the presence of increasing concentrations of *Piper* genus-derived compounds. The results have been obtained in four independent experiments.

## Supplementary Materials

The following are available online at https://www.mdpi.com/2072-6694/11/9/1336/s1, Figure S1: NMR spectroscopy (400 MHz, CDCl_3_) and HRESIMS of compound 1, Figure S2: NMR spectroscopy (400 MHz, CDCl_3_) and HRESIMS of compound 2, Figure S3: NMR spectroscopy (400 MHz, CDCl_3_) and HRESIMS of compound 3, Figure S4: NMR spectroscopy (400 MHz, CDCl_3_) and HRESIMS of compound 4, Figure S5: NMR spectroscopy (400 MHz, CDCl_3_) and HRESIMS of compound 5, Figure S6: Western blot, Figure S7: Protein-ligand interactions fraction for evaluated ligands and X-linked inhibitor of apoptosis protein (XIAP) baculovirus IAP repeat (BIR)-3 domain during the molecular dynamics trajectory of 50 ns, Table S1: ^1^H NMR (400 MHz) data for compounds 1–4 in CDCl_3_ and compound 5 in MeOD, Table S2: ^13^C NMR (100 MHz) data for compounds 1–4 in CDCl_3_ and compound 5 in MeOD.
