# Supplementary material for: XIAP as a Target of New Small Organic Natural Molecules Inducing Human Cancer Cell Death
Source: Cancers (Basel). 2019 Sep 9;11(9):1336. doi: 10.3390/cancers11091336 (PMC6770071; doi:10.3390/cancers11091336)
Supplement: Supplementary file 1 [file cancers-11-01336-s001.pdf]

Supplementary Materials

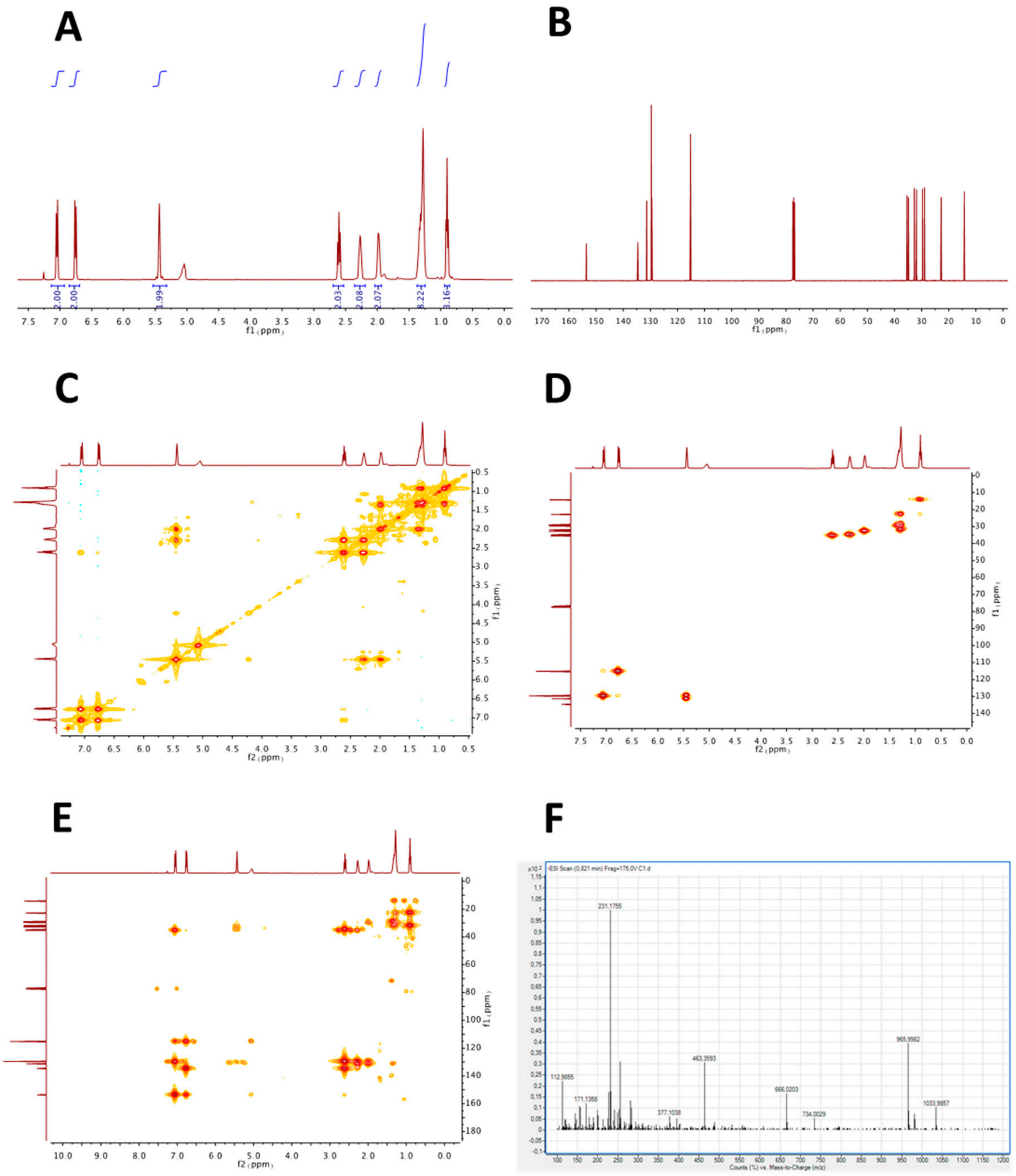

Figure S1. NMR spectroscopy (400 MHz, $CDCl_3$) and HRESIMS of compound 1. (**A**) ^1H NMR spectrum. (**B**) ^{13}C NMR spectrum (75 MHz, $CDCL_3$). (**C**) ^1H-^1H COSY spectrum. (**D**) HMQC spectrum. (**E**) HMBC spectrum. (**F**) HRESIMS spectrum.

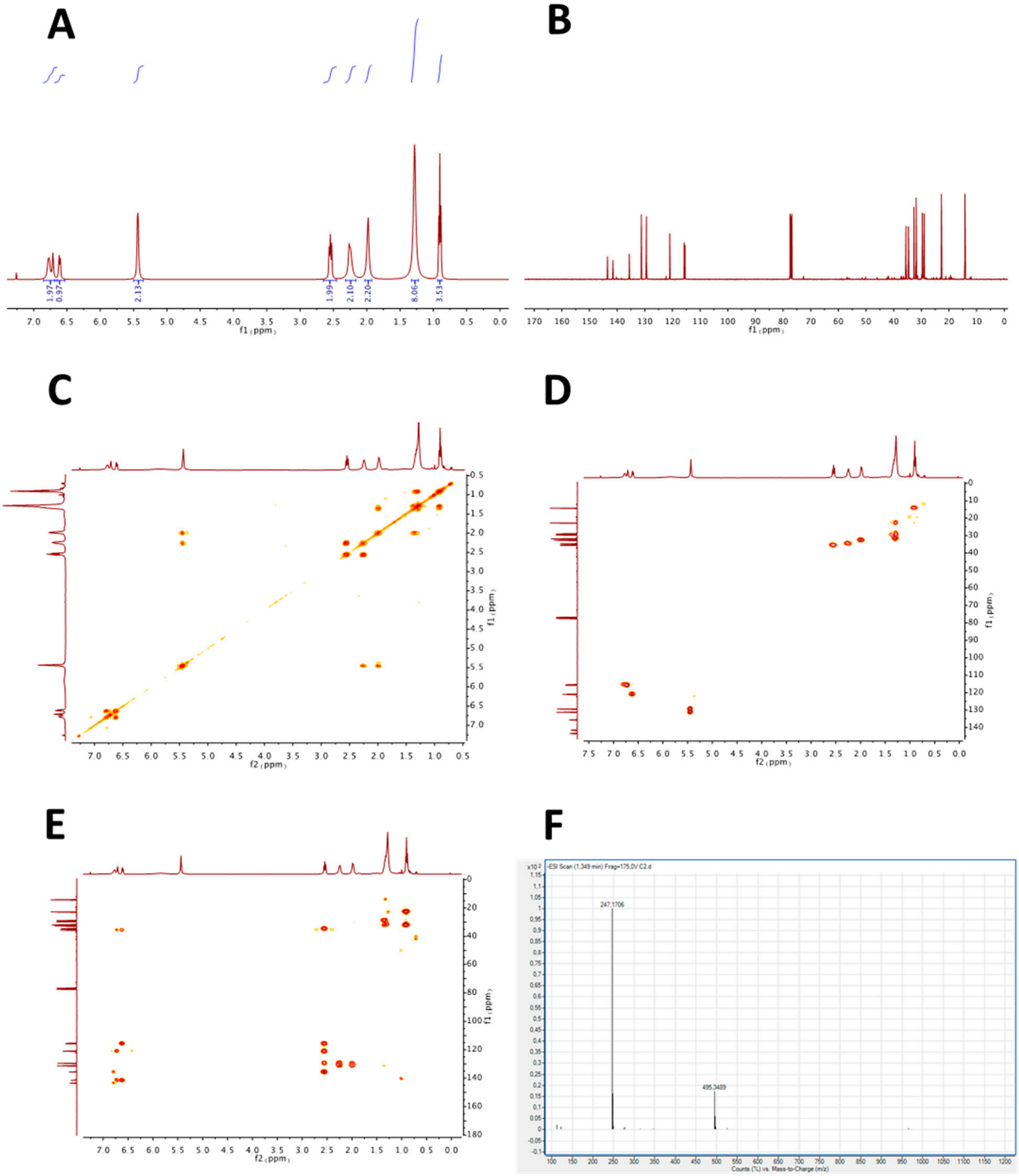

Figure S2. NMR spectroscopy (400 MHz, $CDCl_3$) and HRESIMS of compound 2. (**A**) ^{1}H NMR spectrum. (**B**) ^{13}C NMR spectrum (75 MHz, $CDCL_3$). (**C**) ^{1}H-^{1}H COSY spectrum. (**D**) HMQC spectrum. (**E**) HMBC spectrum. (**F**) HRESIMS spectrum.

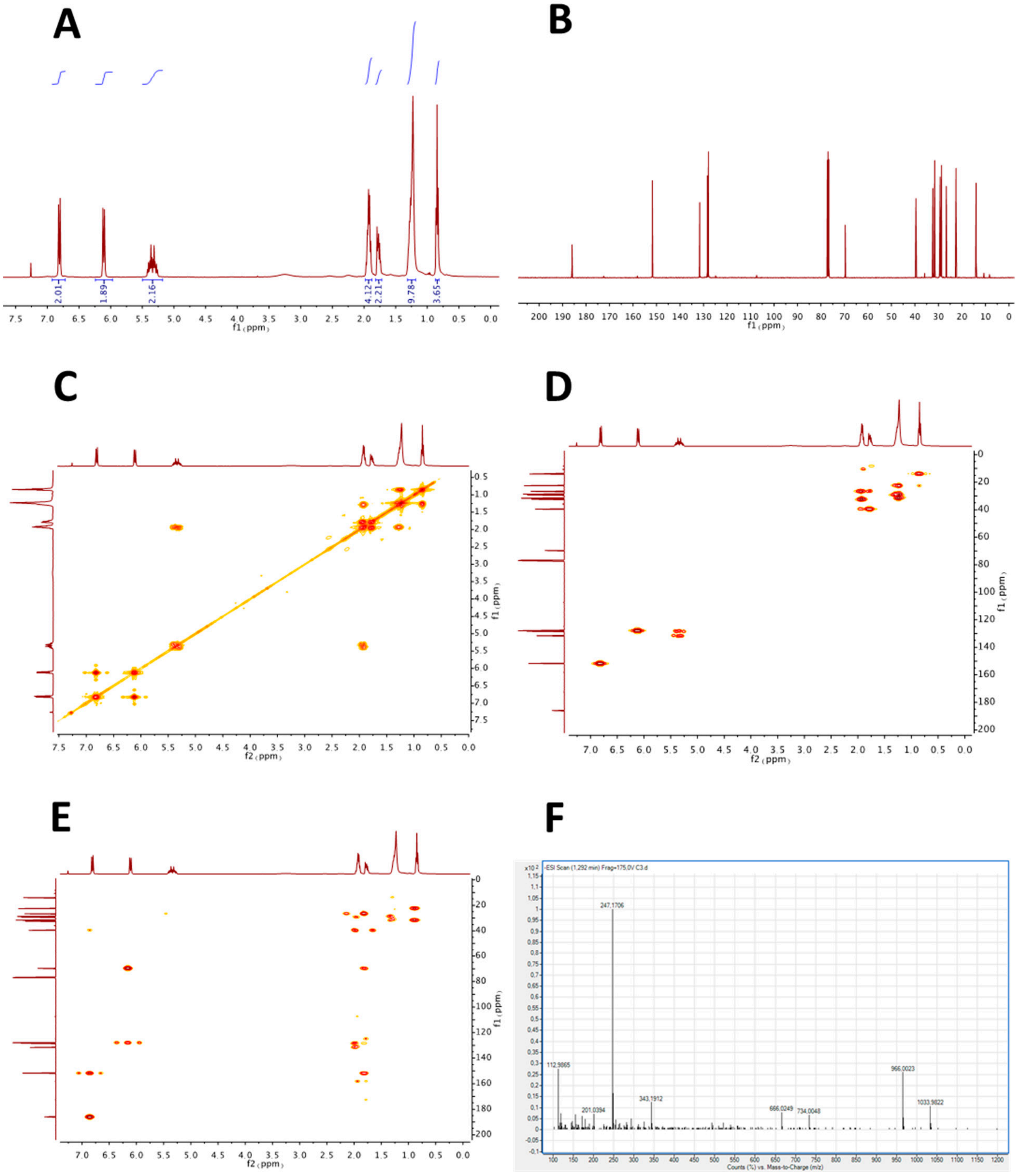

Figure S3. NMR spectroscopy (400 MHz, $CDCl_3$) and HRESIMS of compound 3. (**A**) ^{1}H NMR spectrum. (**B**) ^{13}C NMR spectrum (75 MHz, $CDCL_3$). (**C**) ^{1}H-^{1}H COSY spectrum. (**D**) HMQC spectrum. (**E**) HMBC spectrum. (**F**) HRESIMS spectrum.

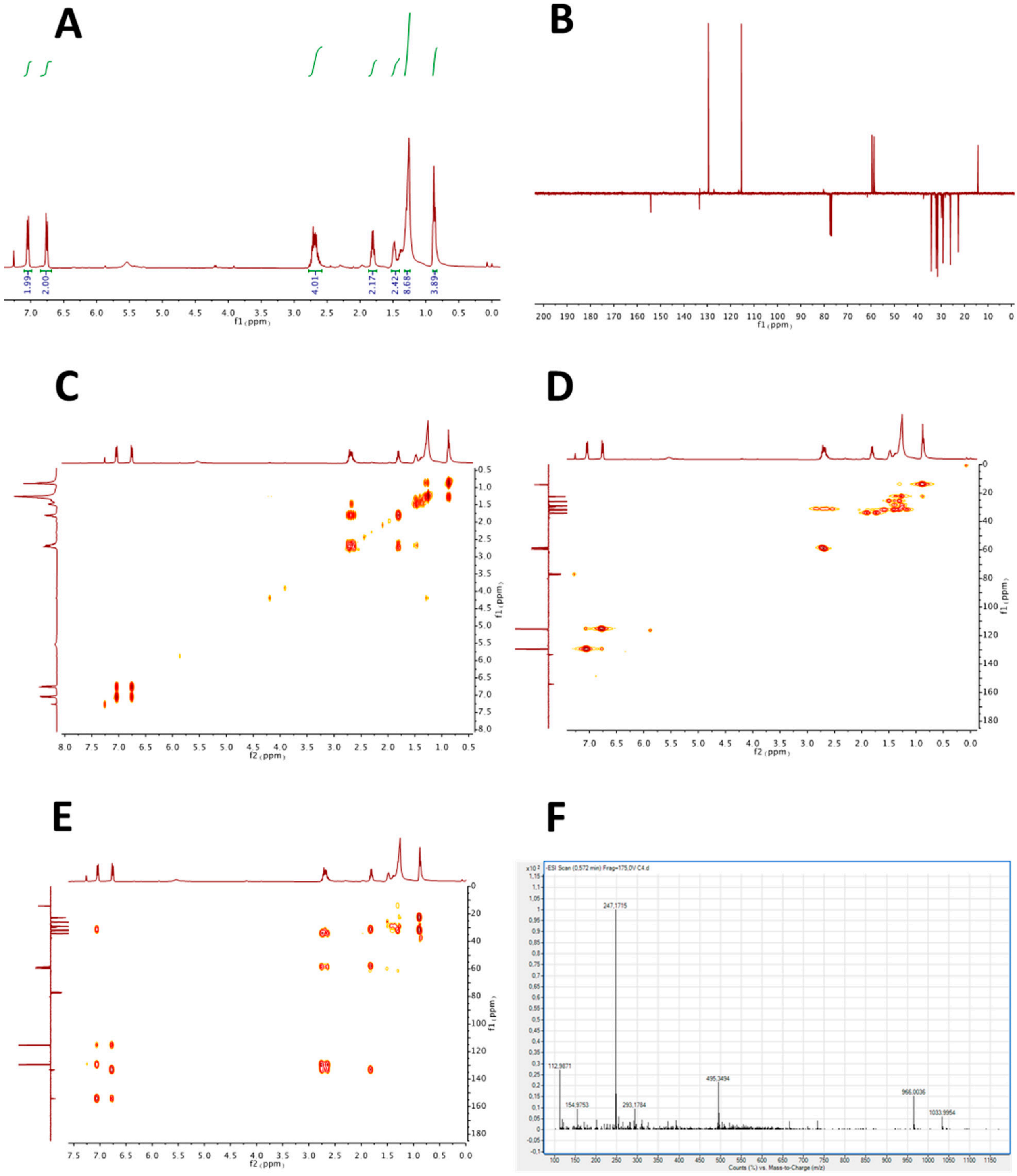

Figure S4. NMR spectroscopy (400 MHz, $CDCl_3$) and HRESIMS of compound 4. (**A**) ^1H NMR spectrum. (**B**) ^{13}C NMR spectrum (75 MHz, $CDCL_3$). (**C**) ^1H-^1H COSY spectrum. (**D**) HMQC spectrum. (**E**) HMBC spectrum. (**F**) HRESIMS spectrum.

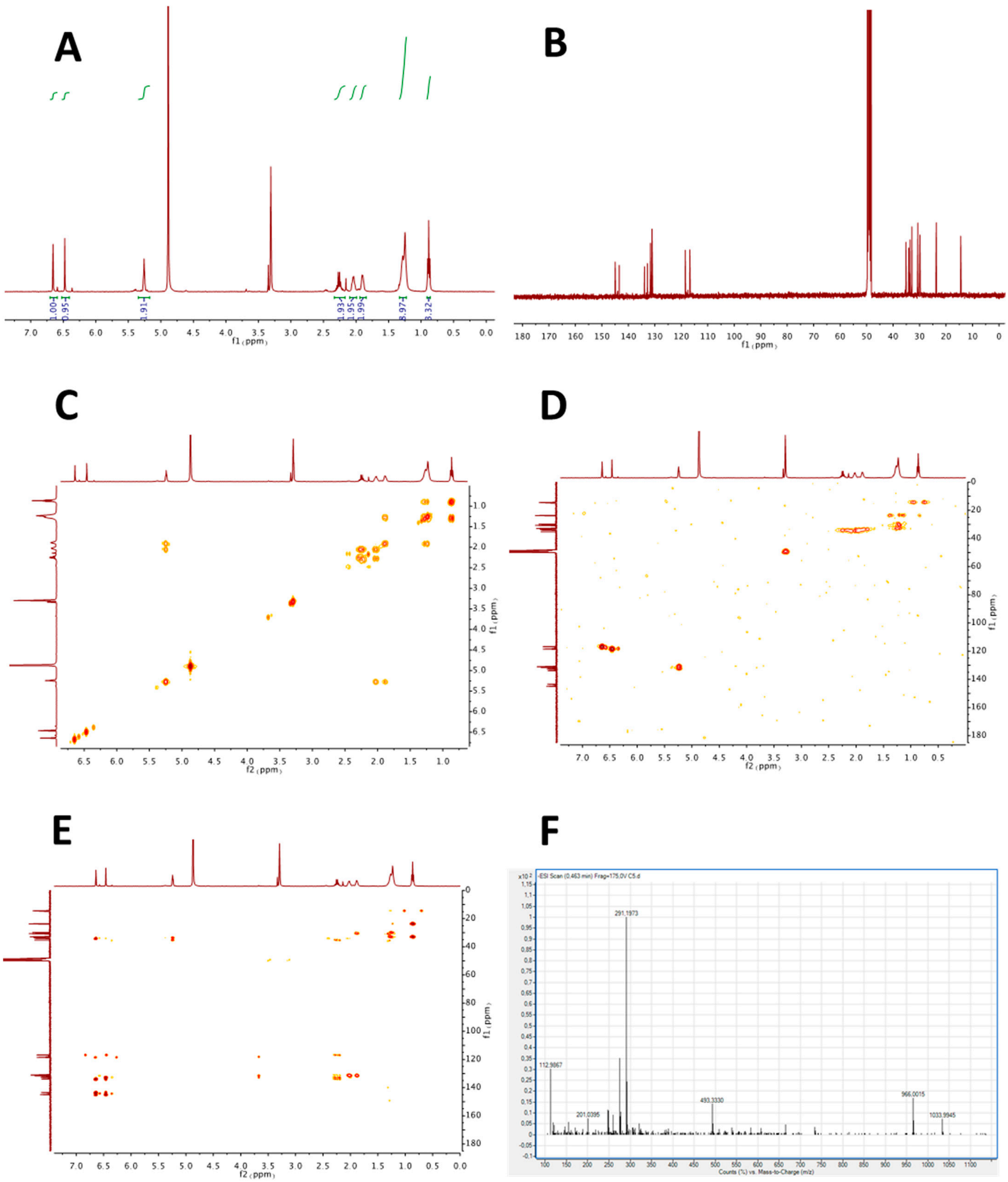

Figure S5. NMR spectroscopy (400 MHz, $CDCl_3$) and HRESIMS of compound 5. (**A**) ^{1}H NMR spectrum. (**B**) ^{13}C NMR spectrum (75 MHz, $CDCL_3$). (**C**) ^{1}H-^{1}H COSY spectrum. (**D**) HMQC spectrum. (**E**) HMBC spectrum. (**F**) HRESIMS spectrum.

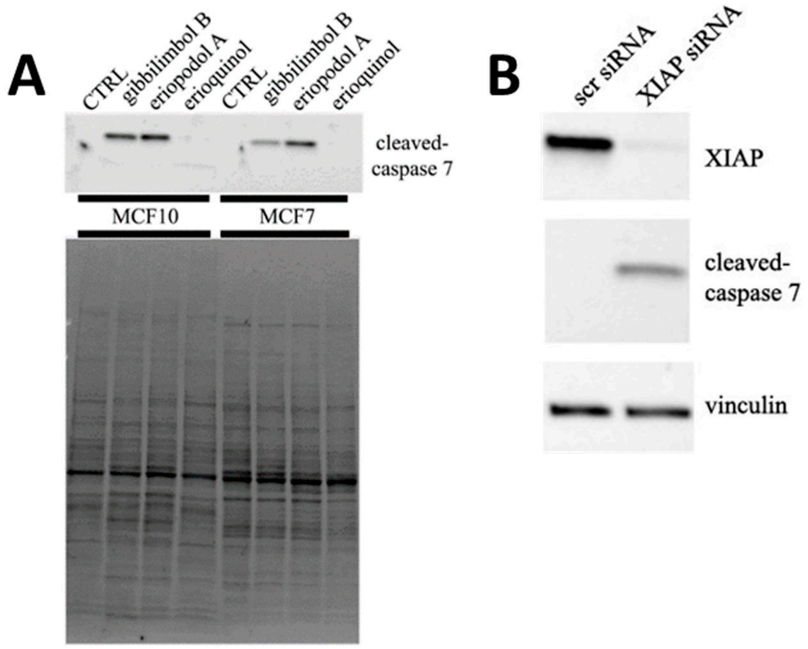

Figure S6. Western blot. (**A**) Western blot analysis of cleaved-caspase 7 in MCF10 and MCF7 cells treated for 6 h in the absence (CTRL, control) and in the presence of 30 μg/mL gibbilimbol B/eriopodol A or 10 μg/mL erioquinol. The stain-free gel was used as loading control. Images are representative of three independent experiments. (**B**) Western blot analysis of X-linked inhibitor of apoptosis protein (XIAP) and cleaved-caspase 7 in MCF7 cells transfected for 24 h with a XIAP-specific and scrambled targeting (scr) siRNA (100 nM). Vinculin was used as internal standard. Images are representative of three independent experiments.

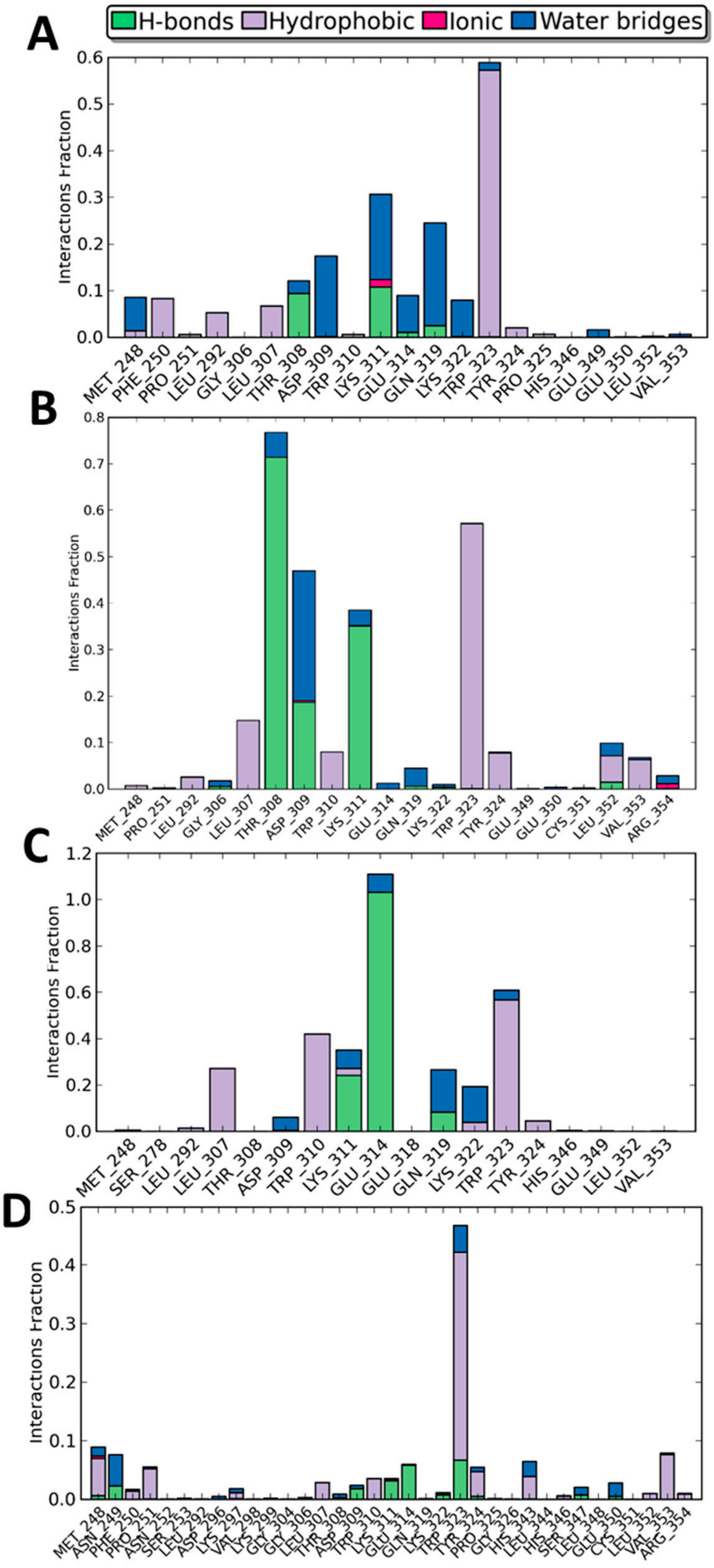

Figure S7. Protein-ligand interactions fraction for evaluated ligands and X-linked inhibitor of apoptosis protein (XIAP) baculovirus IAP repeat (BIR)-3 domain during the molecular dynamics trajectory of 50 ns. (**A**) embelin, (**B**) erioquinol, (**C**) eriopodol A and (**D**) gibbilimbol B.

Table S1. ^{1}H NMR (400 MHz) data for compounds 1–4 in $CDCl_3$ and compound 5 in MeOD.

Position	δH (J in Hz)				
	C-1	C-2	C-3	C-4	C-5
1	-	-	-	-	-
2	6.75 (2H, d, *J* = 8.48)	-	6.11 (2H, d, *J* = 9.94)	6.76 (2H, d, *J* = 8.3)	-
3	7.04 (2H, d, *J* = 8.47)	6.71 (1H, s)	6.81 (2H, d, *J* = 9.96)	7.04 (2H, d, *J* = 8.3)	6.51 (1H, s)
4	-	-	-	-	-
5	7.04 (2H, d, *J* = 8.47)	6.60 (1H, d, *J* = 7.5)	6.81 (2H, d, *J* = 9.96)	7.04 (2H, d, *J* = 8.3)	-
6	6.75 (2H, d, *J* = 8.48)	6.77 (1H, d, *J* = 7.6)	6.11 (2H, d, *J* = 9.94)	6.76 (2H, d, *J* = 8.3)	6.69 (1H, s)
1′	2.60 (2H, t, *J* = 7.3)	2.54 (2H, t, *J* = 7.3)	1.77 (2H, m)	2.69 (2H, m)	2.26 (2H, m)
2′	2.26 (2H, m)	2.24 (2H, m)	1.93 (2H, m)	1.81 (2H, m)	2.04(2H, m)
3′	5.42 (2H, brs)	5.42 (2H, brs)	5.34 (2H, m)	2.69 (1H, m)	5.26 (1H, brs)
4′	5.42 (2H, brs)	5.42 (2H, brs)	5.34 (2H, m)	2.69 (1H, m)	5.26 (1H, brs)
5′	1.97 (2H, m)	1.98 (2H, m)	1.93 (2H, m)	1.48 (2H, m)	1.91 (2H, m)
6′	1.26 (2H, m)	1.27 (2H, m)	1.23 (2H, m)	1.27 (2H, m)	1.29 (2H, m)
7′	1.26 (2H, m)	1.27 (2H, m)	1.23 (2H, m)	1.27 (2H, m)	1.29 (2H, m)
8′	1.26 (2H, m)	1.27 (2H, m)	1.23 (2H, m)	1.27 (2H, m)	1.29 (2H, m)
9′	1.26 (2H, m)	1.27 (2H, m)	1.23 (2H, m)	1.27 (2H, m)	1.29 (2H, m)
10′	0.89 (3H, t, *J* = 6.8)	0.89 (3H, t, *J* = 6.7)	0.85 (3H, t, *J* = 6.75)	0.88 (3H, t, *J* = 6.5)	1.29 (2H, m)
11′	-	-	-	-	1.29 (2H, m)
12′	-	-	-	-	0.89 (3H, t, *J* = 6.8)

Table S2. ^{13}C NMR (100 MHz) data for compounds 1–4 in $CDCl_3$ and compound 5 in MeOD.

Position	δC (ppm)				
	C-1	C-2	C-3	C-4	C-5
1	153.4	141.3	185.9	154.1	144.9
2	115.0	143.4	128.3	115.4	143.3
3	129.5	115.6	151.7	129.5	118.4
4	134.4	135.5	69.6	133.3	132.7
5	129.5	120.8	151.7	129.5	133.9
6	115.0	115.3	128.3	115.4	116.8
1′	35.2	35.4	39.5	31.5	34.1
2′	34.6	34.6	32.4	34.2	35.1
3′	129.2	129.3	127.9	59.6	131.0
4′	131.1	131.1	131.6	58.6	131.6
5′	32.5	32.6	26.6	32.1	33.5
6′	29.5	29.5	29.3	29.2	29.8
7′	28.8	28.8	28.7	26.0	30.6
8′	31.7	31.7	31.6	31.8	30.6
9′	22.6	22.6	22.5	22.6	32.8
10′	14.0	14.1	14.0	14.1	32.8
11′	-	-	-	-	23.6
12′	-	-	-	-	14.4
